# Influence of Structural Features of Peptides on Their Affinity to Cotton Linters Paper

**DOI:** 10.1021/acsomega.5c10906

**Published:** 2026-01-16

**Authors:** Lukas Robert Blawert, Katja Schmitz

**Affiliations:** Biological Chemistry, Department of Chemistry, Technical University of Darmstadt, Darmstadt 64278, Germany

## Abstract

As an alternative to chemical functionalization of cellulose, fusion constructs with carbohydrate-binding modules (CBMs) can be used for the noncovalent immobilization of various compounds and functionalities on cellulose. Smaller cellulose-binding peptides might be used as an alternative, as they are easy to modify and can be produced completely synthetically. To investigate which structural features of peptides promote binding to cotton linters paper, we have established a label-free assay to assess paper affinity. Even though tyrosine residues are essential for the binding of CBMs, we found that the Y/A exchange in peptides did not lead to a reduction in the affinity. This confirms previous assumptions that aromatic structures that are needed to orient CBMs during cellulose binding are less important for smaller, more flexible peptides. We also show that the aromatic fluorophore 5(6)-carboxytetramethylrhodamine (TAMRA), which is sometimes used for peptide labeling, leads to an increase in affinity due to an avidity effect. In addition, we observed that peptides with a *C*-terminal carboxylate group or carboxylate side chains have a lower affinity, and peptides with positively charged amino groups have a higher affinity than the corresponding uncharged peptides. We attributed this to electrostatic interactions with carboxylate groups on the paper. While most peptides tested in this study bound with *K*_D_ values in the midmicromolar range, the combination of a *C*-terminal amide and an *N*-terminal TAMRA modification yielded a peptide with affinity to cotton linters paper in the low micromolar range. The findings presented in this work confirm and expand findings from previous work and reveal limitations and features that point the way toward high-affinity peptides for the functionalization of cellulose.

## Graphical abstract

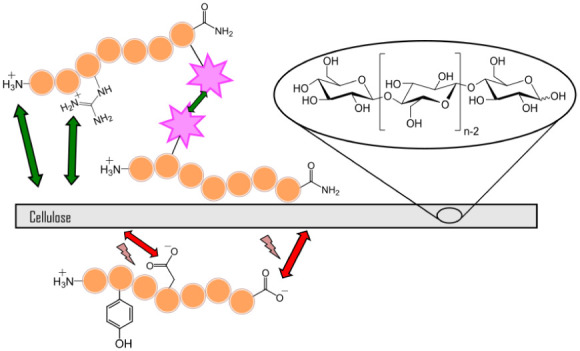

## Introduction

Nowadays, cellulose-based materials are becoming increasingly important, partly because cellulose is the most abundant biopolymer in the biosphere and is easily accessible.^1,2^ For many applications, cellulose must be chemically modified in order to conjugate the bioactive compounds or introduce the desired functionalities.^3^ An alternative to chemical modification is the noncovalent modification of cellulose by paper-binding biomolecules conjugated with the compound to be immobilized. Such conjugates can be used in medical applications, e.g., in lateral flow assays, wound dressings, or for enzyme immobilization.^4–6^

Carbohydrate-binding modules (CBMs) are natural protein domains for recognizing and binding carbohydrate chains.^6^ The CBMs consist of 30–200 amino acids. Most of them have no catalytic activity and are often linked to enzymes, which hydrolytically degrade carbohydrate chains.^6–8^ Tyrosine and tryptophan, which form CH-π interactions with the CH groups of the polysaccharide structure, were found to be essential for cellulose binding.^8–10^ Likewise, polar side chains and the backbone amide bonds interact with the polar groups of the polysaccharide through hydrogen bonds.^11^

CBMs can be easily obtained by heterologous expression.^12^ Selective modification can be accomplished by amber suppression, enzymatic methods, or residue-specific chemical methods. However, compounds produced by genetically engineered organisms may be subject to additional regulatory steps during the approval and production of medicinal products. At the same time, chemical functionalities can be selectively incorporated into peptides, or they can be readily bioconjugated during synthesis. For this reason, fully synthetic, short cellulose-binding peptides are an alternative to larger CBMs for the modification of cellulose. In order to identify sequences for such peptides, phage display libraries were created by Serizawa et al. and Guo et al. and two classes of heptapeptides were identified that bind to nanocrystalline cellulose (NCC) with high affinity.^13,14^ On the one hand, hydroxyl- and amino group-containing peptides were found as good binders, on the other hand, peptides rich in aliphatic side chains were enriched during phage display.^13,14^ Another approach is to derive short peptide sequences from CBMs. For example, Khazanov et al. developed a cyclic peptide from the binding sequence of Cel7A-CBM1, which binds to NCC with low micromolar affinity.^15^ In our own work, we found that even shorter linear peptides from the Cel7A-CBM1 sequence bind to NCC with comparable affinity.^16^ While the results of Reinikainen et al. had shown that tyrosine residues are essential for binding in the context of the larger CBMs, tyrosine-alanine exchange did not reduce affinity to NCC in the short peptide sequences investigated by Lill et al.^9,16^

In the work presented here, the influence of individual structural elements in peptide sequences on binding to cellulose was investigated. For this purpose, we established an HPLC-based label-free method to determine dissociation constants. This method operates with cotton linters paper, which better reflects the later use of peptides for paper modification. As opposed to the previously used fluorescence polarization assay, which works only for soluble binding partners, our label-free method operates with solid cellulose. This allowed us to determine the influence of peptide labeling with the dye 5(6)-carboxytetramethylrhodamine (TAMRA), which was used in previous works by Lill et al.^16^

As model peptides, a phage display-derived peptide (PDDP) was compiled based on the results of Serizawa et al., and a cellulose-binding peptide (CelBP) was derived from Cel7A-CBM1.^14^ We also included the apoptotically active peptide (AAP) described by Friedl et al., as well as the peptide SM1 from the work of Lill et al., which was initially designed to stabilize histone deacetylase 4.^16–18^ These two sequences were used to check whether short peptides that are not known for their cellulose binding also have an affinity for this material. By exchanging amino acids and modifying the termini, we investigated the influence of aromatic side chains and the impact of electrostatic interactions of the termini and charged amino acid side chains on binding to cellulose. Thus, we could confirm that tyrosine residues are not essential for cellulose binding of peptides. Furthermore, we postulate that electrostatic interactions with acidic groups of the cellulose determine the affinity of the peptide.

## Materials and Methods

### Chemicals and Materials

All amino acids, diisopropylcarbodiimide (DIC), and hydroxyiminocyanoacetic acid ethyl ester (Oxyma) were purchased from Carbolution Chemicals GmbH (St. Ingbert, DEU). Diisopropylethylamine (DIPEA), acetic anhydride, dimethylformamide, and dichloromethane (DCM) were from Carl Roth GmbH + Co. KG (Karlsruhe, DEU). Acetonitrile was obtained from Thermo Fisher Scientific (Waltham, USA), trifluoroacetic acid (TFA) from VWR International (Darmstadt, DEU), anisole from Sigma-Aldrich (Merck KGaA, Darmstadt, DEU), and triisopropylsilane (TIPS) from TCI Chemicals (Tokyo, JPN). Nanocrystalline cellulose was purchased from CelluForce Inc. (Montréal, CAN), cotton linters paper was obtained from PD Dr. Tobias Meckel at TU Darmstadt, the peptide CelBP-TAMRA from Dr. Annika Lill, and 5(6)­carboxytetramethylrhodamine (TAMRA) was synthesized by Dr. Kevin Brahm according to Kvach et al.^16,19^ Both are former members of our working group.

### Peptide Synthesis

The peptides were prepared manually by solid-phase peptide synthesis (SPPS) using the standard Fmoc/tBu strategy. Oxyma and DIC were used as coupling reagents. For activation, mixtures of amino acids and the respective coupling reagents were incubated for 10 min, then added to the resin and shaken for 1 h at 600 rpm at room temperature. This was repeated once. 2-Chlorotrityl-chloride resin (1.211 mmol/g, Carbolution, St. Ingbert, DEU) was used to prepare peptides with a free *C*-terminus and Fmoc-Rink-Amide-AM resin (0.722 mmol/g, Carbolution, St. Ingbert, DEU) for peptides with an amide functionality at the *C*-terminus. For acetylation, 50 equiv of DIPEA and 50 equiv of acetic anhydride in dimethylformamide were used and reacted for 60 min on the shaker.

For incorporation of TAMRA into the lysine side chain, the building block Fmoc-Lys­(Mtt)–OH was incorporated, and subsequently, the methyltrityl (Mtt) protecting group on the amino group was selectively cleaved with 5% TFA, 5% TIPS in DCM. 5(6)-Carboxy-TAMRA was coupled to the deprotected lysine side chain using the same procedure as that for amino acid coupling. To cleave the peptide from the resin, 95% TFA, 1.25% TIPS, 1.25% anisole, and 2.5% ultrapure water were added, and the mixture was shaken for 4 h at 600 rpm. The peptides were purified by reverse-phase high-performance liquid chromatography (HPLC) (Shimadzu, Kyoto, JPN) with two LC20AD pumps and an SPD-M20A photodiode array detector. A YMC-Triart Prep C18–S column (250 mm × 10 mm, 10 μm) (YMC, Kyoto, JPN) was employed, and the peptide was eluted with a gradient of acetonitrile (ACN) and ultrapure water with 0.1% TFA. To determine the purity, the peptides were analyzed by reversed-phase HPLC and mass spectrometry. The chromatograms and absorption spectra are shown in the Supporting Information (SI).

### Analytical HPLC

A YMC Triart C18 column (150 mm × 4.6 mm, S 5 μm, 12 nm) (YMC, Kyoto, JPN) was used for the analytical sample runs. For elution, 95% ultrapure water, 5% ACN + 0.1% TFA (eluent A) and 5% ultrapure water, 95% ACN + 0.1% TFA (eluent B) were used at a flow rate of 1.5 mL/min. The proportion of eluent B was increased from 0% to 100% between 1.0–8.5 min, then the column was rinsed with eluent B until 11.5 min, and the fraction of eluent B was reduced to 0% until 12.0 min. The column was equilibrated with eluent A for 3 min. The injection volume was 10 μL. If there was no linear dependence between area and concentration during peptide quantification at higher concentrations, only 1 μL was injected, and the results were calculated using the dilution factor. All measurements used for quantification were performed in technical triplicates. The core sequences and retention times of the investigated peptides are shown in Table 1.

**Table 1:** Sequences and Retention Times of the Model Peptides with the *C*-Terminal Amide

Peptide	Sequence	t_R_ (min)
PDDP	H-SQTLYAR-NH_2_	4.51
CelBP	H-GQVLNPYYSQCK-NH_2_	4.87
SM1	H-GSITQGIPR-NH_2_	4.71
AAP	H-RAYVVM-NH_2_	5.06

### Affinity Determination of Peptides to Cotton Linters Paper

To determine affinity, peptide solutions were incubated with paper discs, and the unbound peptides from the supernatant were quantified. We initially attempted to determine the free peptide concentration by a bicinchoninic acid (BCA) assay. However, we observed a high background value for untreated paper. When we analyzed supernatants from peptide solutions incubated with paper by HPLC with UV/vis detection, we found an unidentified compound eluting at short retention times that absorbs at the absorption maximum of the unlabeled peptides. We assumed that this compound is released from the paper and is likely responsible for the high background values in the BCA assay. For this reason, HPLC was necessary to separate the interfering compound from the actual peptide, and the UV/vis detector was used for quantification at the respective absorption maximum of the peptide.

All peptides were dissolved in ultrapure water. To create a calibration curve, the solutions of a dilution series were each analyzed three times by HPLC as described above, and the average area of the peak at the respective absorption maximum of the peptide was plotted against the concentration. With these average areas and the calibration curve, the initial concentrations of the solutions before adsorption (c_0_) could be determined at the same time.

60 μL of each of the differently concentrated peptide solutions were incubated with Whatman filter papers, grade 43 (Merck KGaA, Darmstadt, DEU), cut into discs with a diameter of 0.5 cm, in a sealed 96-well microtiter test plate for 4 h at 23 °C.

The papers were then removed, the microtiter test plate was centrifuged at 2194 g for 5 min to remove lose fibers, and then the supernatant of each well was analyzed in triplicates using HPLC to determine the concentration of unbound peptide at equilibrium (c_free_). The molar amount of peptide bound to the paper (n_bound_) was determined from the difference between c_0_ and c_free_ and plotted against c_free_. The data points were fitted by nonlinear regression in GraphPad Prism (version 9.5, GraphPad Software, LLC) using the “Specific binding with Hill slope” (eq 1). The coefficient h describes the cooperativity of the binding.

1 $$n_{\mathrm{bound}}=\frac{B_{\max}\times c_{\mathrm{free}}^{h}}{K_{D}^{h}\times c_{\mathrm{free}}^{h}}$$

### Circular Dichroism (CD)

A Jasco J-1500 CD spectrometer (Jasco Inc., Easton, USA) was used for the CD spectroscopy measurements. These were performed in a quartz glass cuvette (Starna Scientific GmbH, Pfungstadt, DEU) with a diameter of 1 mm at a temperature of 20 °C in the wavelength range of 190–260 nm, with a scan rate of 50 nm/min and a step size of 0.2 nm. In each case, 10 measurements were carried out, and the values obtained for each measurement point were averaged. The concentration of the investigated peptide solutions was 25 μM. When measuring with NCC, the concentration of the peptides was 25 μM and that of the NCC was 8 μM. Ultrapure water was used as the solvent. When measuring the peptide solutions with NCC, the mixture was incubated for 1 h. A blank was subtracted from each measurement, either ultrapure water or 8 μM NCC in ultrapure water. The raw data were converted to mean residue ellipticity (MRE) using CDToolX software (Birkbeck College, University of London, London, GBR).^20^ The CD spectra of all peptides are shown in the Supporting Information.

### Determination of the Acid Group Loading on Cellulose

100 mg portion of the cotton linters paper was suspended in 65 mL of ultrapure water, and then 5 mL of 0.01 M NaCl and 1 mL of 0.1 M HCl are added. A conductivity titration of the suspension was carried out using a Titrando 905 instrument (Metrohm, Herisau, CHE) and a conductivity module 856 instrument (Metrohm, Herisau, CHE). Every 10 s, 0.02 mL of 0.01 M NaOH solution was titrated up to a final volume of the base of 2 mL. The conductivity was plotted against the volume of NaOH, and a straight line A was drawn through the data points between data point 100 and the point 30 data points before the minimum, and a straight line B was drawn between the thirtieth data point after the minimum up to data point 950. A horizontal line C was plotted tangentially to the conductivity minimum. The number of acid groups was determined from the intersection points V_1_ and V_2_ of the lines A and B with C according to eq 2 Ultrapure water was titrated as a reference to obtain the reference intersection points of the reference *V*_1‑ref_ and *V*_2‑ref_.

2 $$\frac{n\left(\mathrm{Acid}\right)}{m\left(\mathrm{Cellulose}\right)}=\frac{c_{NaOH}\times \left(V_{2}-V_{1}-\left(V_{2-\mathrm{Ref}}-V_{1-\mathrm{Ref}}\right)\right)}{m\left(\mathrm{Cellulose}\right)}$$

## Results and Discussion

### Design of a Cellulose Binding Sequence

To determine the influence of individual structural elements in peptide sequences on binding to cellulose, we designed a model peptide based on the findings of Serizawa et al. They had shown, by phage display, that cellulose-binding sequences often contain hydroxyl-containing amino acids in positions 1 and 5 and a basic amino acid in position 7.^14^ For the remaining positions, the amino acids Q, T, L, and A were selected according to frequently occurring amino acids in the cellulose-binding sequences, resulting in the sequence H-SQTLYAR-NH_2_. We termed this as phage display-derived peptide.

### Interactions of the Tyrosine Side Chains

It has been described that the tyrosine side chains are essential for the binding of CBMs to cellulose.^21^ These aromatic structures are aligned parallel to the cellulose fiber, resulting in the formation of CH-π interactions.^8,10^ However, in our own group, we have shown that for short peptide sequences, the exchange of tyrosine residues does not lead to a decrease in affinity, suggesting that these interactions are not essential for binding.^16^

To further assess the influence of the aromatic ring structure, as well as that of the entire side chain, the tyrosine in the PDDP sequence was replaced by serine and by alanine, respectively. The K_D_-values of all three peptides were in the midmicromolar range (see Table 2).

**Table 2:** Dissociation Constants and Hillslopes of the Nonlinear Regression of PDDP and Its Homologues Binding to Cotton Linters Paper^a^

Peptide	Sequence	*K*_D_ (μM)	Hillslope
PDDP	H-SQTLYAR-NH_2_	28.4 ± 6.4	0.8 ± 0.1
PDDP-Y5S	H-SQTLSAR-NH_2_	14.6 ± 3.2	1.1 ± 0.2
PDDP-Y5A	H-SQTLAAR-NH_2_	35.7 ± 10.9	0.9 ± 0.3

a “Specific binding with Hill slope” equation was used for fitting the data in GraphPad Prism.

All three peptides showed a similar hillslope of approximately 1. The starting sequence of PDDP, as well as PDDP-Y5A, showed a comparable affinity toward cotton linters paper. This indicates that the tyrosine side chains in linear peptides have no influence on the binding affinity of the peptide, confirming the observation of Lill et al.^16^ Viegas et al. hypothesized that the tyrosine side chains of CBMs play an important role in the guiding and packing of the carbohydrate chain, whereas side chain residues of aspartic acid, serine, arginine, or glutamic acid, which can form hydrogen bonds, are important for the binding of the substrate.^11^

In their work, Viegas et al. refer to larger proteins with a more rigid structure.^11^ The CD spectrum in Figure 1 shows that the peptides described in Table 2 are unstructured and, therefore, flexible. It is possible that the more flexible peptides do not require orientation by the tyrosine side chains, as the flexible shorter peptide structure can more easily attach to the cellulose surface. This could explain why the Y/A exchange leads to a loss of affinity in larger proteins. No significant change in affinity was observed by the Y/A exchange in the peptide PDDP. When replacing the tyrosine in PDDP with a serine, a decrease in the dissociation constant from 28.4 μM to 14.6 μM was achieved, corresponding to an increase in affinity. This may be due to the fact that serine is more flexible and can therefore better position its hydroxyl group to form hydrogen bonds than the corresponding tyrosine. This supports the hypothesis that flexible peptide structures can attach better to the cellulose surface than those of more rigid proteins.

**Figure 1:**
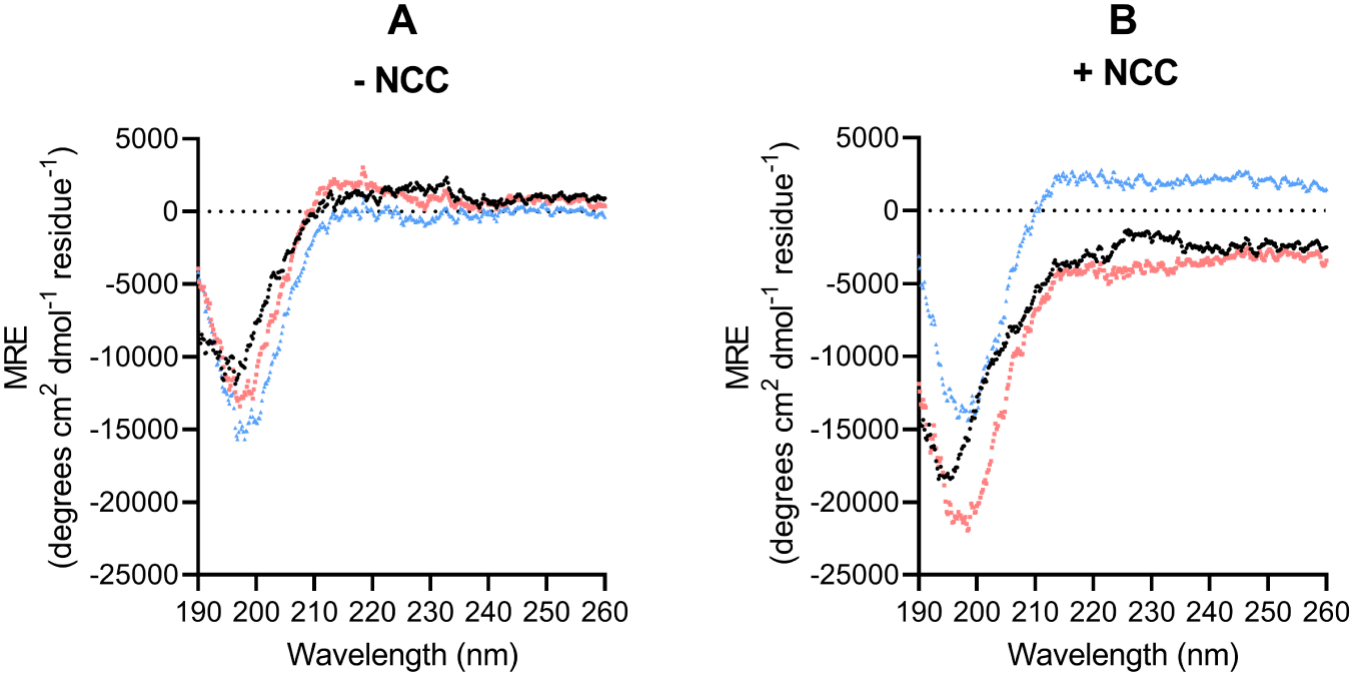
CD spectra of PDDP (black dots), PDDP-Y5S (red squares), and PDDP-Y5A (blue triangles) in ultrapure water. Measurements were made at 20 °C between 190 and 260 nm with a peptide concentration of 25 μM (left) and with the same peptide concentration and NCC at a concentration of 8 μM (right).

The CD spectra of the same peptides in the presence of NCC revealed no structural change upon binding to NCC. This suggests that the binding constant of the three peptides was not influenced by structural changes in the peptide upon binding to cellulose. NCC was used for these experiments because it is not possible to prepare a homogeneous suspension of cotton linters paper. As NCC and cotton linters differ in the amount of amorphous and crystalline regions, the CD spectra presented in this work should be interpreted with caution.

### Influence of TAMRA Labeling

The results of Lill et al., which were obtained with TAMRA-labeled peptides, showed no loss of affinity upon Y/A exchange in the CelBP sequence.^16^ TAMRA is a rhodamine dye with aromatic structures that could form CH-π interactions like tyrosine and thereby compensate for the effect of the Y/A exchange. As the HPLC-based method does not require a label, it was possible to compare the binding of labeled and unlabeled peptides to determine whether the reported affinities were due to TAMRA labeling. The CelBP and the negative control SM1 from the work of Lill et al. as well as the PDDP were used for this purpose.^16^ The affinity of pure TAMRA to cotton linters paper was also examined as a reference. PDDP and SM1 were labeled via the *N*-terminus, and CelBP via the amine of the lysine side chain, because the *N*-terminally labeled CelBP was not soluble at higher concentrations. The determined *K*_D_ values and hillslopes of the nonlinear regression are shown in Figure 2.

**Figure 2:**
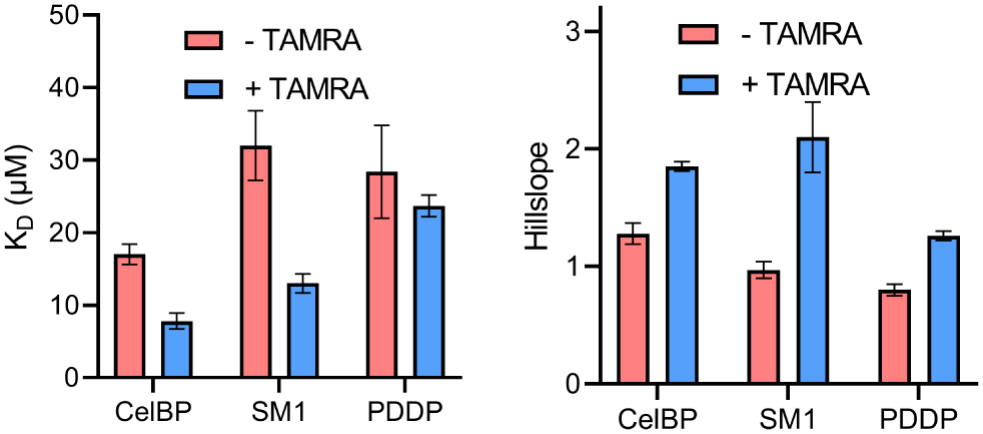
Affinities (left) and hillslopes (right) of the nonlinear regression to investigate the influence of the fluorophore TAMRA by binding to cotton linters paper. “Specific binding with Hill slope” equation was used for fitting the data in GraphPad Prism.

The *K*_D_ values from Figure 2 are all in the lower micromolar range. In contrast, the measured K_D_ values of the TAMRA-labeled SM1 and CelBP peptides from Lill et al. were lower when binding to NCC (0.27–0.62 μM).^16^ We explain the lower dissociation constants measured for NCC compared to cotton linters paper by the different types of surface areas of the cellulose materials.^22–25^ While NCC features predominantly crystalline cellulose, both crystalline and amorphous cellulose are present in cotton linters paper. The fluorescence anisotropy data with NCC suggest that peptides bind to crystalline cellulose with higher affinity than to amorphous cellulose, so that the affinity to paper with both types of surfaces is lower.

All labeled peptides from Figure 2 showed lower *K*_D_ values, i.e., a higher affinity, than the corresponding unlabeled peptides. The pure fluorophore has only a low affinity for cotton linters paper, with a *K*_D_ value of >300 μM (data not shown).

Remarkably, the hillslopes for TAMRA-labeled peptides are higher than those for the corresponding unlabeled peptides. This indicates positive cooperativity in binding, mediated by the fluorophore. The hillslope of the TAMRA-labeled peptides from Lill et al. was also 1.9–2.8.^16^ This can be explained by the aggregation of the fluorophore, which is known to occur with rhodamine dyes.^26,27^ In fact, the labeled peptides, as well as the fluorophore TAMRA, tend to dimerize, as supported by their absorption spectra in Figure 3. The maximum at lower wavelengths that occurs for the labeled peptides and TAMRA can be assigned to the respective dimer, since the relative absorption of this maximum decreases as the concentration decreases (data not shown). The observed positive cooperativity of the labeled peptides can thus be attributed to the fluorophore.

**Figure 3:**
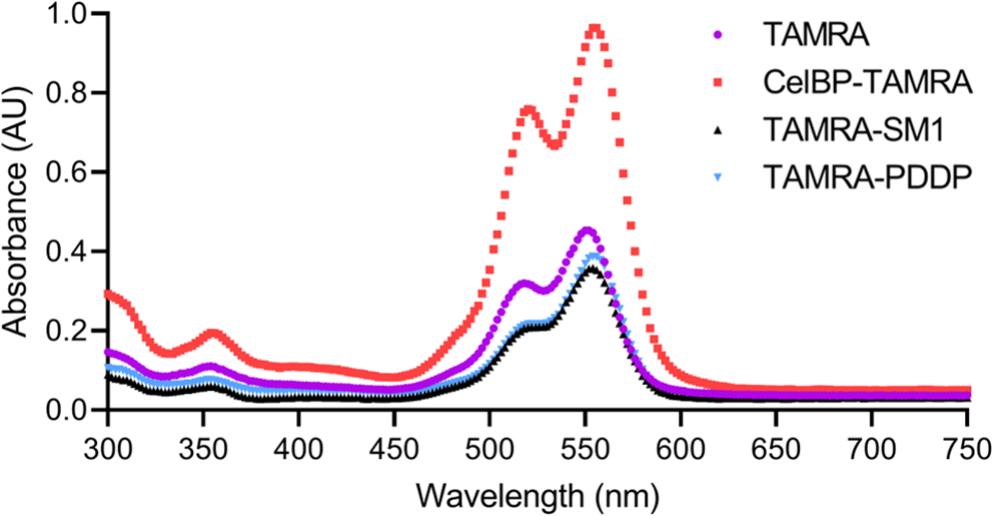
Absorbance spectra of TAMRA (purple), CelBP-TAMRA (H-GQVLNPYYSQCK­(TAMRA)-NH_2_, red), TAMRA-SM1 (TAMRA-GSITQGIPR-NH_2_, black), and TAMRA-PDDP (TAMRA- SQTLYAR-NH_2_, cyan) in ultrapure water, c = 150 μM. The maximum at higher wavelengths can be assigned to the monomer.^27^

When a labeled peptide binds to cellulose and its TAMRA group associates with that of a free peptide, the free peptide is brought into spatial proximity to the cellulose. Thereby, its local concentration and the probability of binding increases. This avidity effect, mediated by the fluorophore, could explain the higher affinity of TAMRA-labeled peptides.

### Electrostatic Interactions

All core sequences tested in this work share a positively charged residue, and Serizawa et al. found that a positively charged amino acid at the *C*-terminus is favorable for cellulose binding.^14^ Viegas et al. argued that some charged side chains may form hydrogen bonds. However, electrostatic interactions of charged groups in a peptide with charged groups of cellulose could influence the binding to cotton linters paper. Therefore, the influence of a free *C*-terminus and side chains with carboxylate groups, as well as that of a free *N*-terminus and side chains with positively charged groups, was examined.

Derivatives of the peptide sequences PDDP, CelBP, AAP, and SM1 were compared, each with a free carboxylate group (indicated by the suffix “-OH”) or an amide group at the *C*-terminus, as well as peptides with a free or acetylated *N*-terminus (indicated by the prefix “Ac-”). Likewise, the effect of charged side chains was investigated by exchanging arginine for alanine and asparagine for aspartic acid. Due to solubility problems, the CelBP could not be prepared with a free carboxyl group at the *C*-terminus or acetylated *N*-terminus so these variants could not be used for the affinity measurements. The dissociation constants of the peptides are listed in Figure 4.

**Figure 4:**
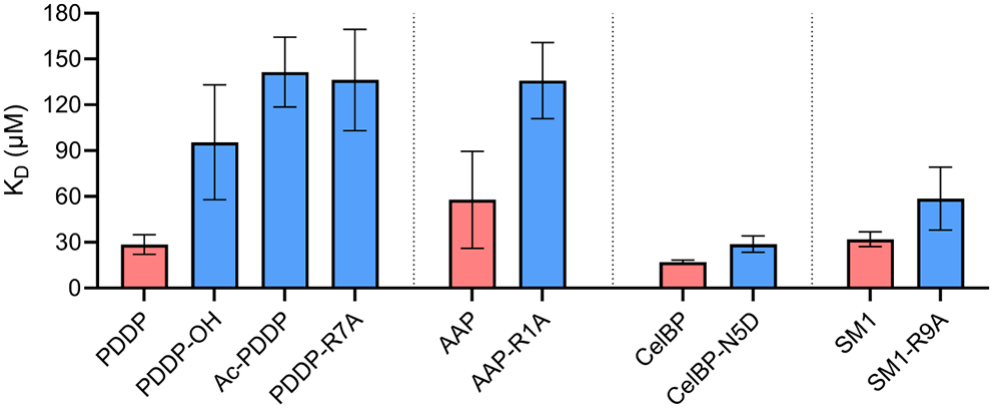
Influence of electrostatic interactions between peptide functionalities and cotton linters paper on dissociation constants. The “Specific binding with Hill slope” equation was used for fitting the data in GraphPad Prism. Peptides with acetylated *N*-terminus are labeled with the prefix “Ac-”, and peptides with free carboxylic acid at the *C*-terminus are labeled with the suffix “-OH”, All other peptides have a free *N*-terminal amino group and *C*-terminal amide. Core sequences are listed in Table 1. Red = core sequence, blue = modified sequence with an additional negative or a positive group. *K*_D_ value of AAP-OH and Ac-AAP > 200 μM.

The data show that the PDDP peptide with a free carboxylate group binds to cellulose more weakly, i.e., with a higher *K*_D_ value, than the corresponding sequences with an amide group at the *C*-terminus. This was confirmed by AAP–OH, for which the *K*_D_ was estimated to be >200 μM (data not shown). This trend can also be observed, although less pronounced, when the charged aspartic acid is replaced by asparagine in the sequence of CelBP. This could be explained by repulsive electrostatic interactions between the negatively charged carboxylate groups of the peptides and those of the cotton linters paper. In fact, acidic groups could be confirmed on the cotton linters paper. For the samples used here, titration of the acid groups against an aqueous sodium hydroxide solution yielded an amount of acidic groups of (0.032 ± 0.007) mmol/g.

The hypothesis that interactions with acid groups of cellulose influence affinity is further supported by the reduced affinities of those peptides, where positive charges were removed. The acetylated peptides exhibit higher dissociation constants, i.e., a lower affinity, than corresponding peptides with a free *N*-terminus. This was confirmed not only by Ac-PDDP but also by Ac-AAP, whose *K*_D_ was estimated to be >200 μM (data not shown). The same applies to the exchange of the positively charged arginine for alanine in the PDDP, AAP, and SM1 sequence. This can be explained by attractive electrostatic interactions between the positively charged groups and the aforementioned acid groups of cellulose. For all tested peptides, neither secondary structures in solution nor the induction of such structures by NCC binding could be detected by CD spectroscopy (see Supporting Information), so that an influence of structural changes on the above findings is unlikely.

## Conclusion and Outlook

In this work, we investigated which structural features of linear peptides influence binding to cotton linters paper. Consistent with previous studies of Lill et al., the replacement of tyrosine with alanine in the heptapeptide sequence PDDP did not lead to a loss of binding affinity.^16^ This confirms that in smaller and more flexible peptides, tyrosine is not essential for paper binding, unlike what has been described for larger proteins.^9^

Our data also suggest that the fluorophore TAMRA, which is frequently used for peptide labeling, does not bind to cellulose itself but causes labeled peptides to dimerize. This leads to higher affinities due to an avidity effect. This is also supported by the positive cooperativity observed in the binding of the labeled peptides.

Moreover, it was found that peptides with a free *C*-terminus and peptides with carboxyl groups in the side chain had a weaker affinity to cotton linters paper than the corresponding sequences with a *C*-terminal amide or amide side chains. In addition, peptides with a free *N*-terminus and positively charged side chains bound better than those with an acetylated *N-*terminus or an uncharged amide group in the side chain. Since acid groups were detected on the paper, these results suggest that negatively charged groups weaken the bond to the paper through electrostatic repulsion, and positively charged ones strengthen the noncovalent binding through electrostatic attraction. None of the peptides analyzed showed a structural change upon binding to NCC. Therefore, a change in the *K*_D_ value is most probably not caused by a structural change upon binding to NCC.

While most peptides tested in this study bound with *K*_D_ values in the midmicromolar range, the combination of a *C*-terminal amide and an *N*-terminal TAMRA modification yielded a peptide with affinity to cotton linters paper in the low micromolar range. It is worth noting that despite the loss of the positive charge of the C-terminal lysine residue, attachment of the rhodamine dye at this position led to an increase in affinity. This deserves further investigation.

The knowledge gained in this work contributes to the understanding of the binding behavior of short peptide sequences on cotton linters paper and the influence of labeling with TAMRA and possibly other rhodamine dyes. As modifications of the termini have a large impact on affinity, the use of the presented peptides as anchoring units for other functionalities on paper is limited. In light of the impact of electrostatic interactions on affinity, peptides with more positively charged side chains might compensate for the effect of terminal modifications. This requires further investigation. Moreover, the roles of backbone flexibility and sequence length also need to be investigated. This will reveal how the affinity of peptides to paper can be increased and to what extent the impact of terminal modifications can be overcome by the core sequence in order to act as a noncovalent anchoring moiety for paper functionalization.

## Supplementary Material



## Notes

MS spectra of the purified peptides, their HPLC chromatograms, and the corresponding UV/Vis spectra. The CD spectra of the peptides in water and with NCC are also shown (PDF)
