# Supplementary material for: Influence of Structural Features of Peptides on Their Affinity to Cotton Linters Paper
Source: ACS Omega. 2026 Jan 16;11(4):6184–90. doi: 10.1021/acsomega.5c10906 (PMC12878760; doi:10.1021/acsomega.5c10906)
Supplement: Supplementary file 1 [file ao5c10906_si_001.pdf]

Supporting Information

Influence of structural features of peptides on their affinity to cotton linters paper

Lukas Robert Blawert[1], Katja Schmitz[1]*

[1] Biological Chemistry, Department of Chemistry, Technical University of Darmstadt, 64278 Darmstadt, Germany

*e-mail: katja.schmitz@tu-darmstadt.de

Table of Contents

1. Association curves of peptide binding to cotton-linters paper 3
2. MS spectra of the purified peptides 13
3. HPLC chromatograms of the purified peptides 23
4. UV/Vis spectra of the purified peptides 35
5. Overview of purities, retention times and absorption maxima of the peptides 46
6. CD spectra of the purified peptides 48

1. Association curves of peptide binding to cotton-linters paper

The workflow for determining the binding affinity of peptides to cotton linters paper is shown in Figure S 1.

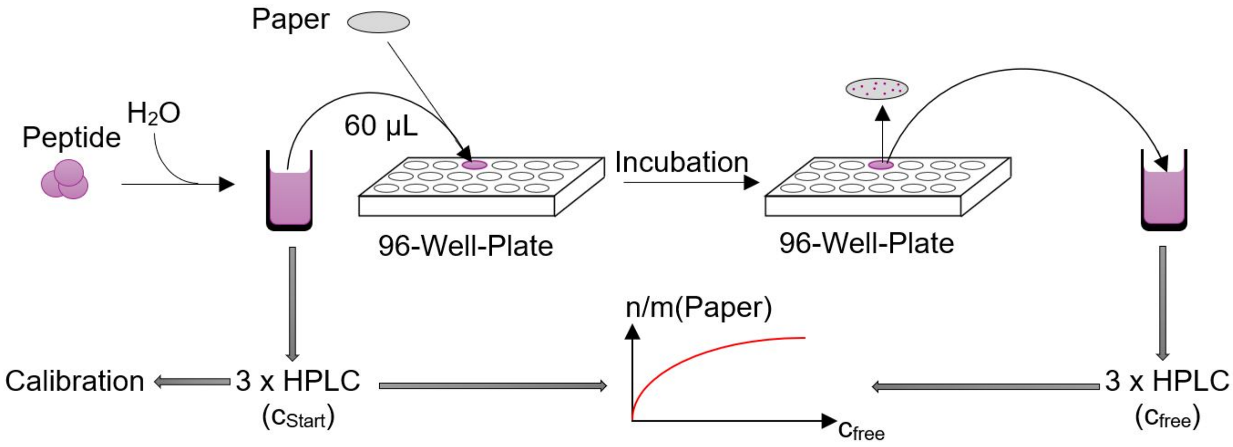

Figure S 1. Workflow for determining the binding affinity of peptides to cotton linters paper.

A calibration series was used to determine the concentration of the peptide solutions. The regression coefficient (R^2_{cali}) for each linear regression is given in the caption of the respective adsorption isotherm. Technical replicas (n = 3) were measured in this process of calibration.

All data from the adsorption isotherms were fitted using the "Specific binding with Hill slope" equation (1) in GraphPad Prism (Version 9.5.0). Technical replicas (n = 3) were measured. The error bars represent one standard deviation.

$$n_{bound} = \frac{B_{max} * c_{free}^{h}}{K_D^h + c_{free}^{h}} \tag{1}$$

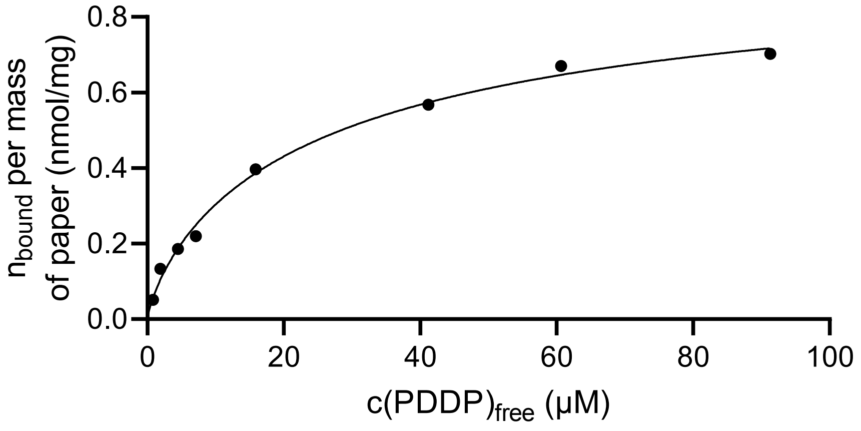

Figure S 2. Adsorption isotherm of PDDP (H-SQTLYAR-NH_2) to cotton-linters-paper. Fitting parameters: K_D = 28.4 ± 6.4 µM, h = 0.8 ± 0.1, B_{max} = 1.00 ± 0.08 nmol/mg, R^2 = 0.994, R^2_{cali} = 0.999.

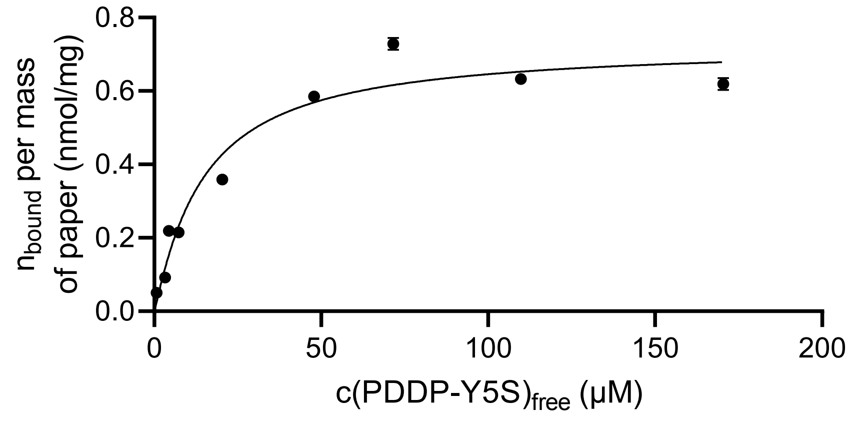

Figure S 3. Adsorption isotherm of PDDP-Y5S (H-SQTLSAR-NH_2) to cotton-linters-paper. Fitting parameters: K_D = 14.6 ± 3.2 µM, h = 1.1 ± 0.2, B_{max} = 0.73 ± 0.05 nmol/mg, R^2 = 0.947, R^2_{cali} = 0.999.

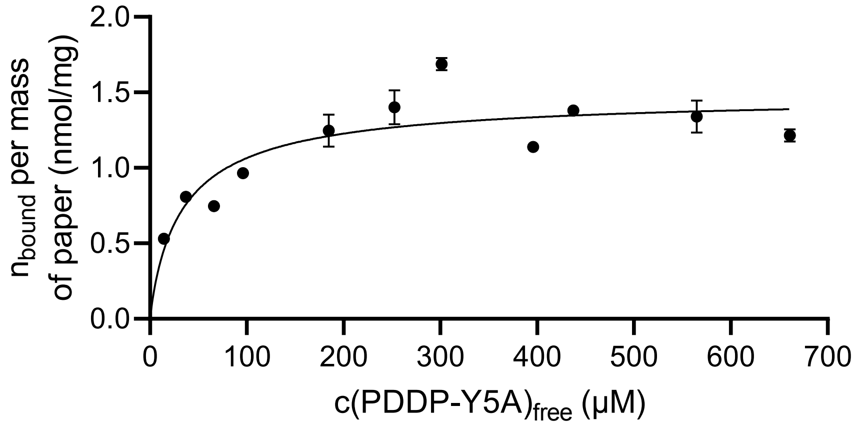

Figure S 4. Adsorption isotherm of PDDP-Y5A (H-SQTLAAR-NH_2) to cotton-linters-paper. Fitting parameters: K_D = 35.7 ± 10.9 µM, h = 0.9 ± 0.3, B_{max} = 1.49 ± 0.15 nmol/mg, R^2 = 0.725, R^2_{cali} = 0.999.

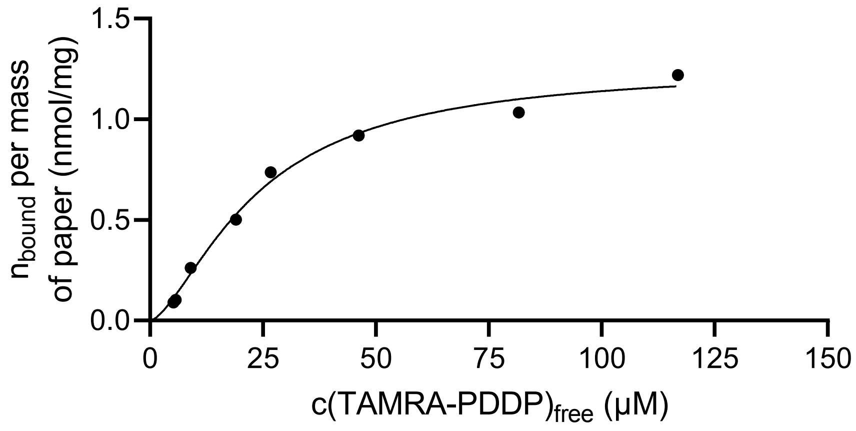

Figure S 5. Adsorption isotherm of TAMRA-PDDP (TAMRA-SQTLYAR-NH_2) to cotton-linters-paper. Fitting parameters: K_D = 23.7 ± 1.5 µM, h = 1.5 ± 0.1, B_{max} = 1.26 ± 0.04 nmol/mg, R^2 = 0.990, R^2_{cali} = 0.999.

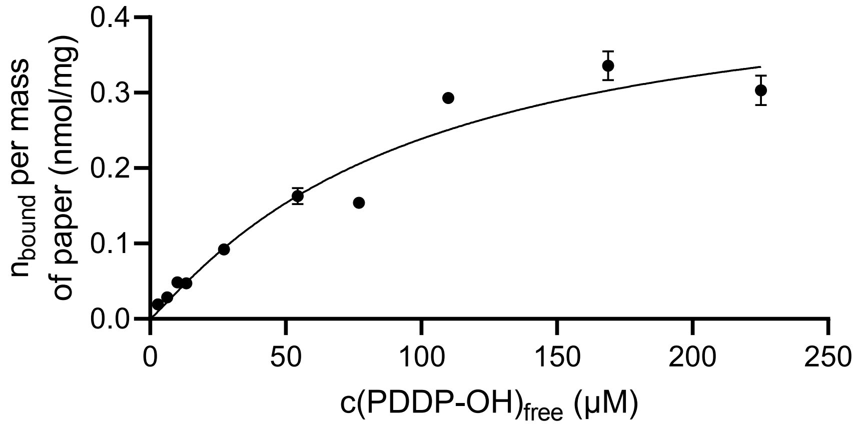

Figure S 6. Adsorption isotherm of PDDP-OH (H-SQTLYAR-OH) to cotton-linters-paper. Fitting parameters: $K_D = 94.5 \pm 37.6$ µM, $h = 1.1 \pm 0.2$, $B_{max} = 0.46 \pm 0.09$ nmol/mg, $R^2 = 0.945$, $R^2_{cali} = 0.999$.

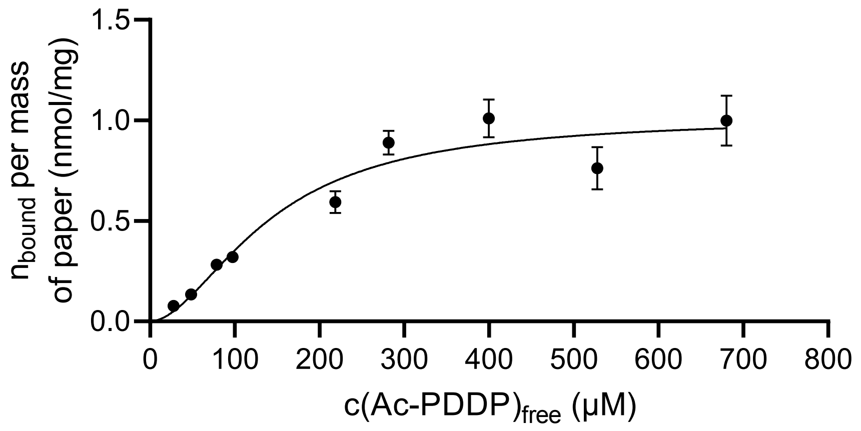

Figure S 7. Adsorption isotherm of Ac-PDDP (Ac-SQTLYAR-NH_2) to cotton-linters-paper. Fitting parameters: $K_D = 141.5 \pm 22.9$ µM, $h = 1.8 \pm 0.4$, $B_{max} = 1.02 \pm 0.09$ nmol/mg, $R^2 = 0.914$, $R^2_{cali} = 0.997$.

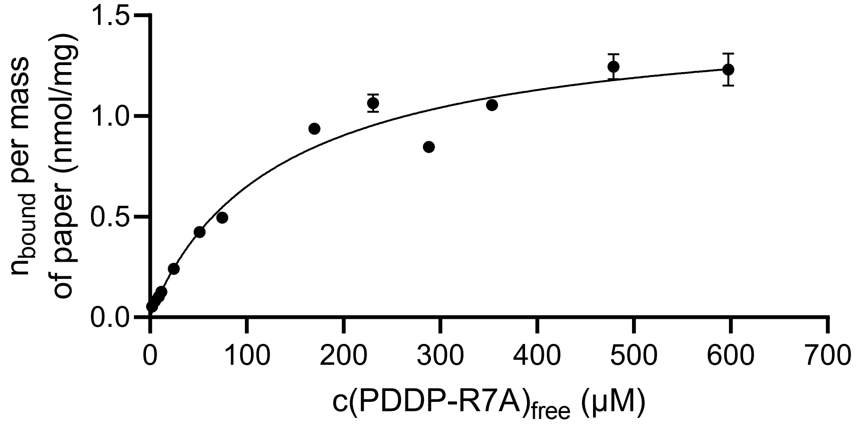

Figure S 8. Adsorption isotherm of PDDP-R7A (H-SQTLYAA-NH_2) to cotton-linters-paper. Fitting parameters: K_D = 136.3 ± 33.1 µM, h = 1.0 ± 0.1, B_{max} = 1.53 ± 0.14 nmol/mg, R^2 = 0.972, R^2_{cali} = 0.999.

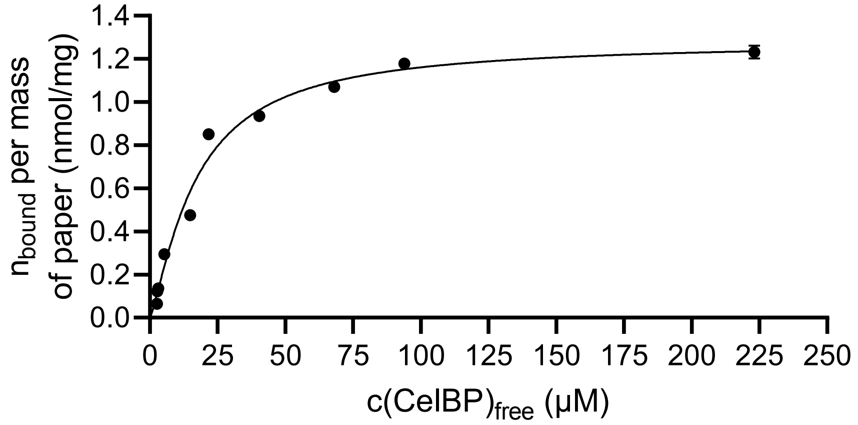

Figure S 9. Adsorption isotherm of CelBP (H-GQVLNPYYSQCK-NH_2) to cotton-linters-paper. Fitting parameters: K_D = 17.0 ± 1.4 µM, h = 1.3 ± 0.1, B_{max} = 1.28 ± 0.04 nmol/mg, R^2 = 0.983, R^2_{cali} = 0.996.

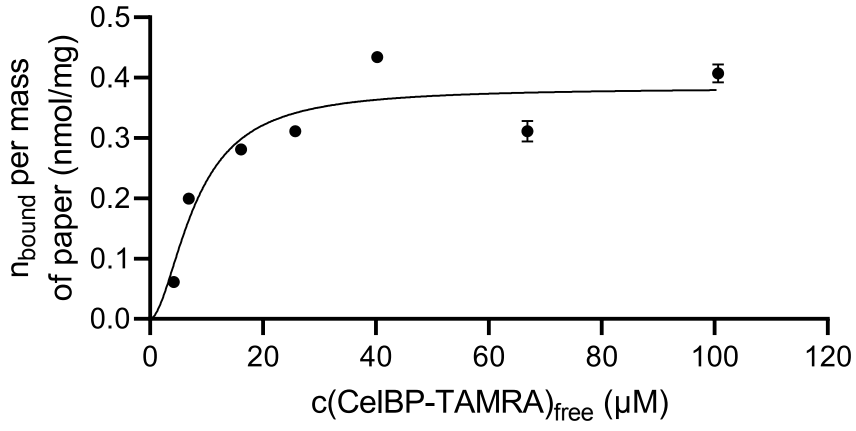

Figure S 10. Adsorption isotherm of CelBP-TAMRA (H-GQVLNPYYSQCK(TAMRA)-NH_2) to cotton-linters-paper. Fitting parameters: K_D = 8.1 ± 1.2 µM, h = 1.8 ± 0.4, B_{max} = 0.38 ± 0.02 nmol/mg, R^2 = 0.860, R^2_{cali} = 0.998.

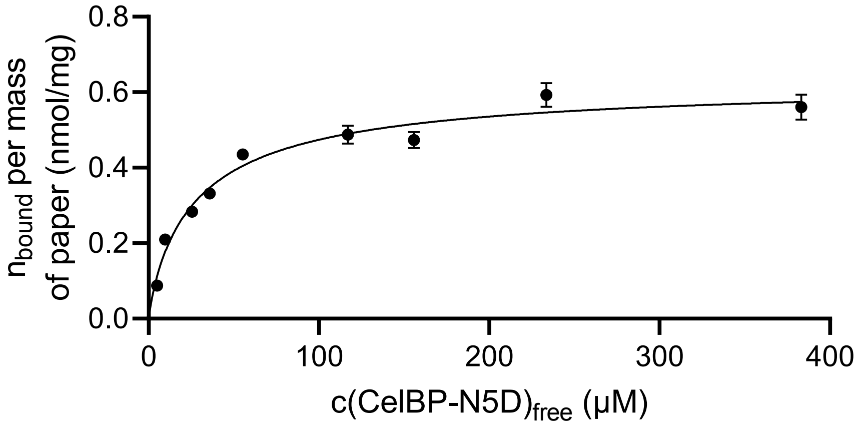

Figure S 11. Adsorption isotherm of CelBP-N5D (H-GQVLDPYYSQCK)-NH_2) to cotton-linters-paper. Fitting parameters: K_D = 28.8 ± 5.3 µM, h = 0.9 ± 0.1, B_{max} = 0.63 ± 0.04 nmol/mg, R^2 = 0.958, R^2_{cali} = 0.999.

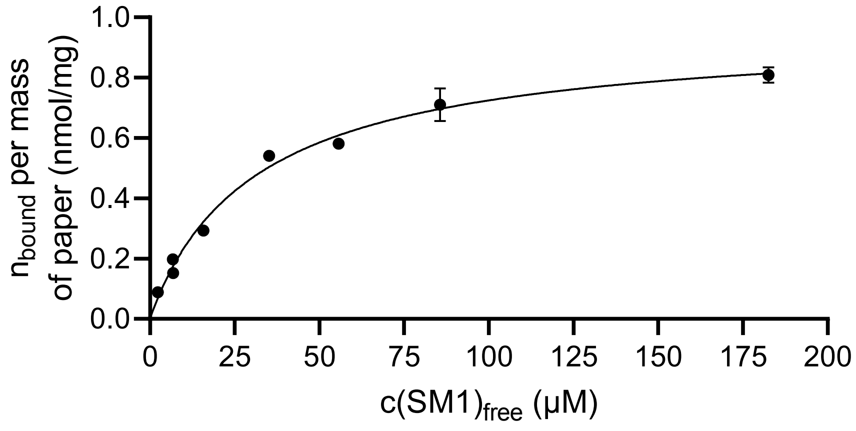

Figure S 12. Adsorption isotherm of SM1 (H-GSITQGIPR-NH_2) to cotton-linters-paper. Fitting parameters: K_D = 32.0 ± 4.8 µM, h = 1.0 ± 0.1, B_{max} = 0.96 ± 0.05 nmol/mg, R^2 = 0.986, R^2_{cali} = 0.999.

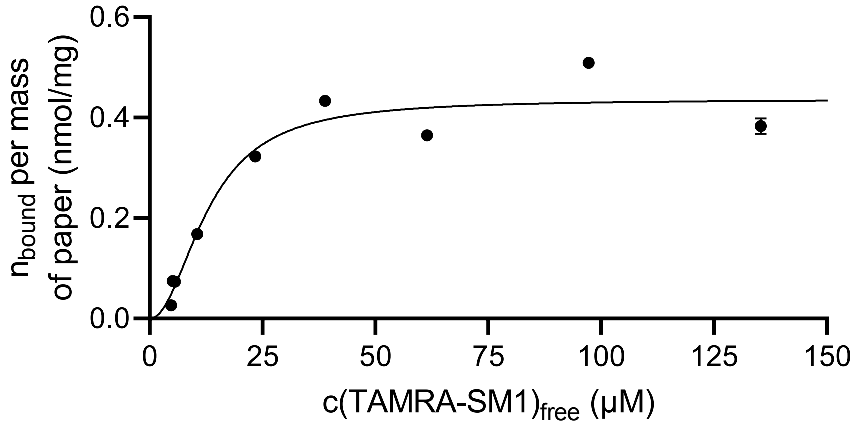

Figure S 13. Adsorption isotherm of TAMRA-SM1 (TAMRA-GSITQGIPR-NH_2) to cotton-linters-paper. Fitting parameters: K_D = 13.0 ± 1.3 µM, h = 2.1 ± 0.3, B_{max} = 0.44 ± 0.02 nmol/mg, R^2 = 0.943, R^2_{cali} = 0.998.

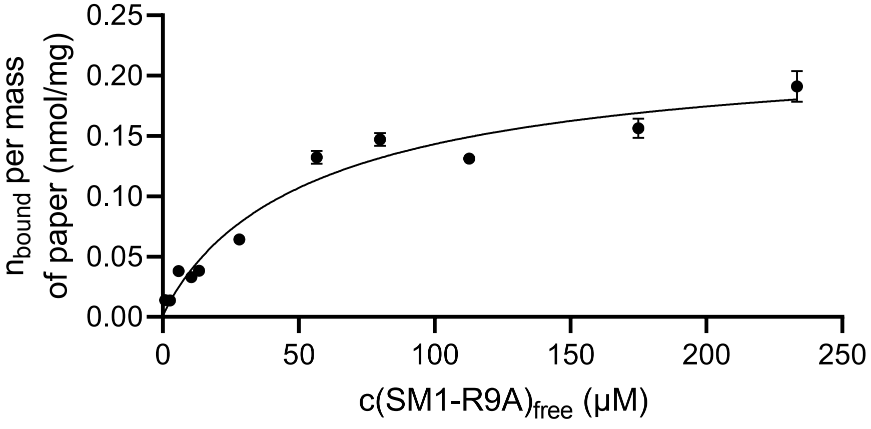

Figure S 14. Adsorption isotherm of SM1-R9A (H-GSITQGIPA-NH_2) to cotton-linters-paper. Fitting parameters: K_D = 58.6 ± 20.6 µM, h = 0.9 ± 0.1, B_{max} = 0.23 ± 0.03 nmol/mg, R^2 = 0.954, R^2_{cali} = 0.999.

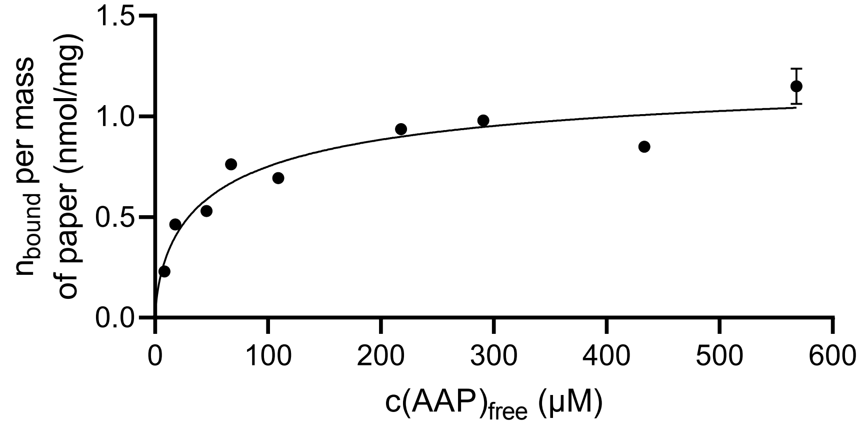

Figure S 15. Adsorption isotherm of AAP (H-RAYVVM-NH_2) to cotton-linters-paper. Fitting parameters: K_D = 57.8 ± 31.8 µM, h = 0.7 ± 0.2, B_{max} = 1.27 ± 0.19 nmol/mg, R^2 = 0.898, R^2_{cali} = 0.999.

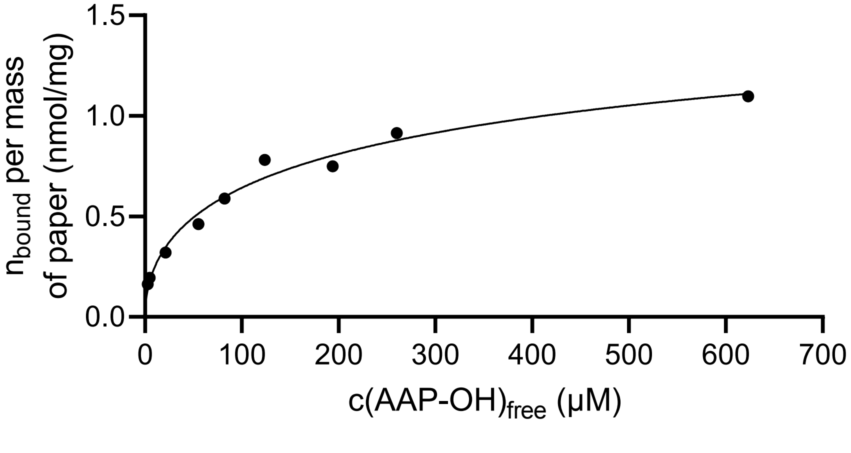

Figure S 16. Adsorption isotherm of AAP-OH (H-RAYVVM-OH) to cotton-linters-paper. Fitting parameters: $K_D > 200$ µM, $h = 0.5 \pm 0.1$, $B_{max} = 2.04 \pm 0.40$ nmol/mg, $R^2 = 0.980$, $R^2_{cali} = 0.997$.

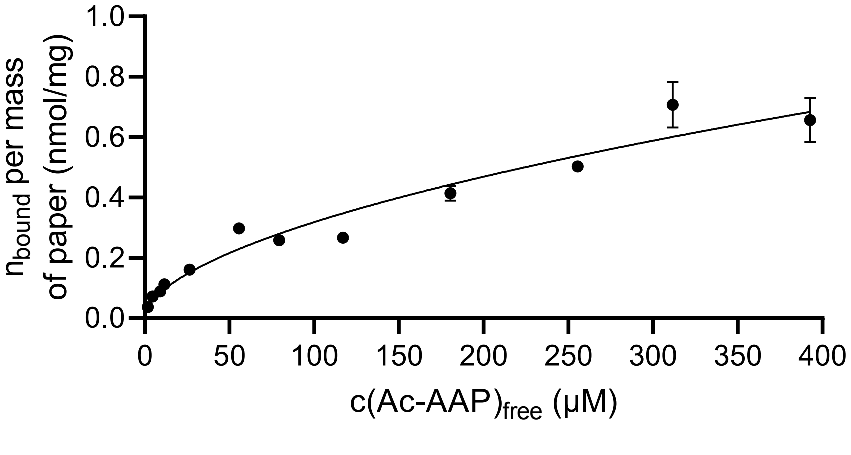

Figure S 17. Adsorption isotherm of Ac-AAP (Ac-RAYVVM-NH_2) to cotton-linters-paper. Fitting parameters: $K_D > 200$ µM, $h = 0.6 \pm 0.1$, B_{max} = not determinable, $R^2 = 0.940$, $R^2_{cali} = 0.999$.

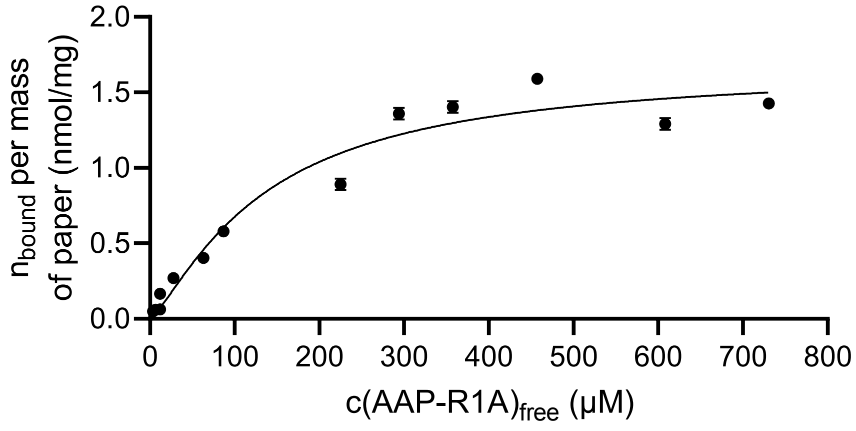

Figure S 18. Adsorption isotherm of AAP-R1A (H-AAYVVM-NH_2) to cotton-linters-paper. Fitting parameters: K_D = 135.9 ± 24.9 µM, h = 1.3 ± 0.2, B_{max} = 1.68 ± 0.13 nmol/mg, R^2 = 0.960, R^2_{cali} = 0.999.

2. MS spectra of the purified peptides

All MALDI-TOF spectra were measured in positive mode; α-cyano-4-hydroxycinnamic acid was used as the sample matrix.

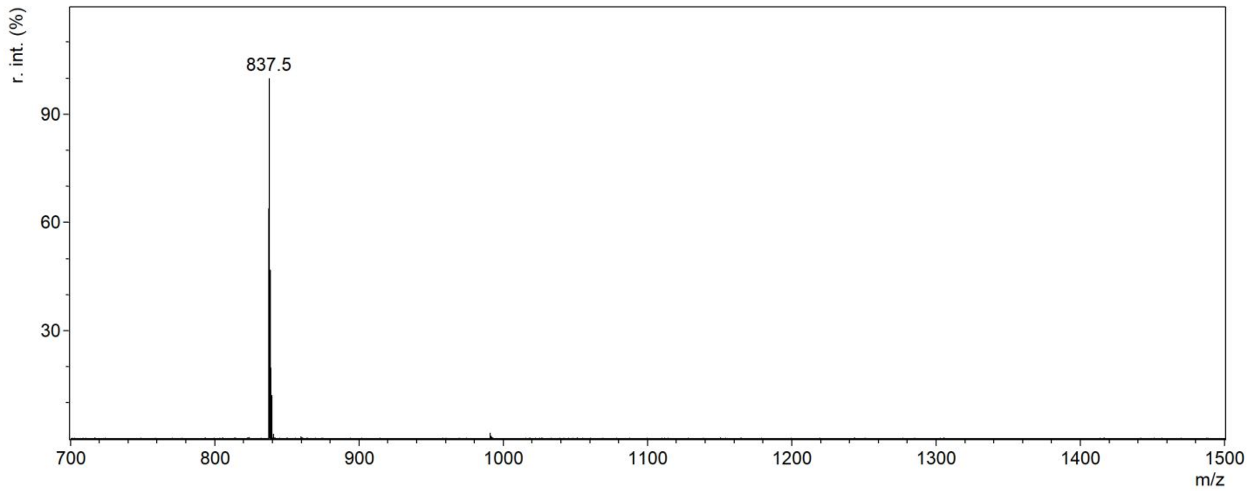

Figure S 19. MALDI-TOF spectrum of purified PDDP (H-SQTLYAR-NH_2) in the range of m/z = 1700-1500. $[M+H]^+$ = 837.5.

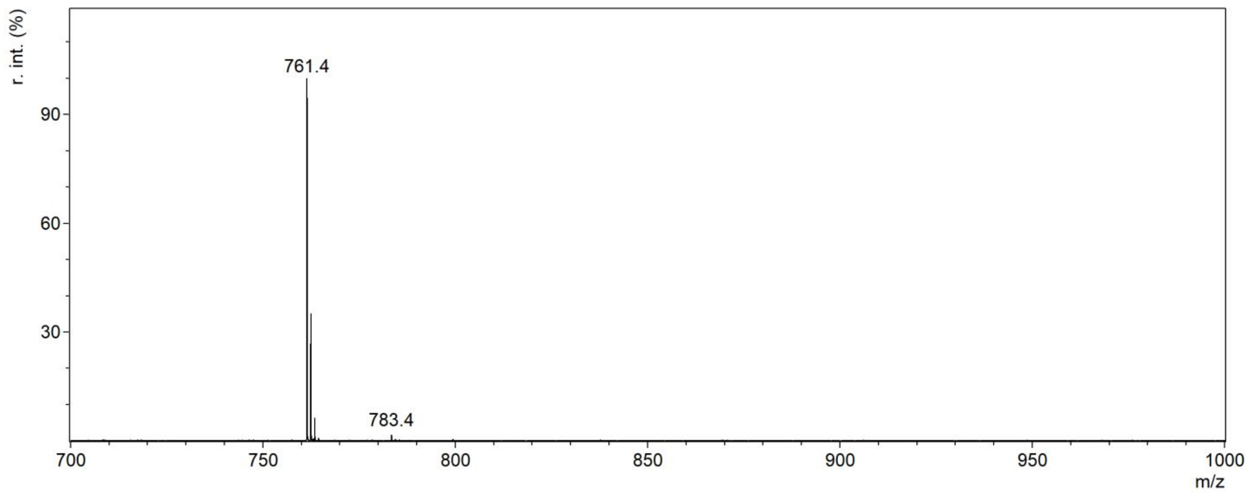

Figure S 20. MALDI-TOF spectrum of purified PDDP-Y5S (H-SQTLSAR-NH_2) in the range of m/z = 700-1000. $[M+H]^+$ = 761.4, $[M+Na]^+$ = 783.4.

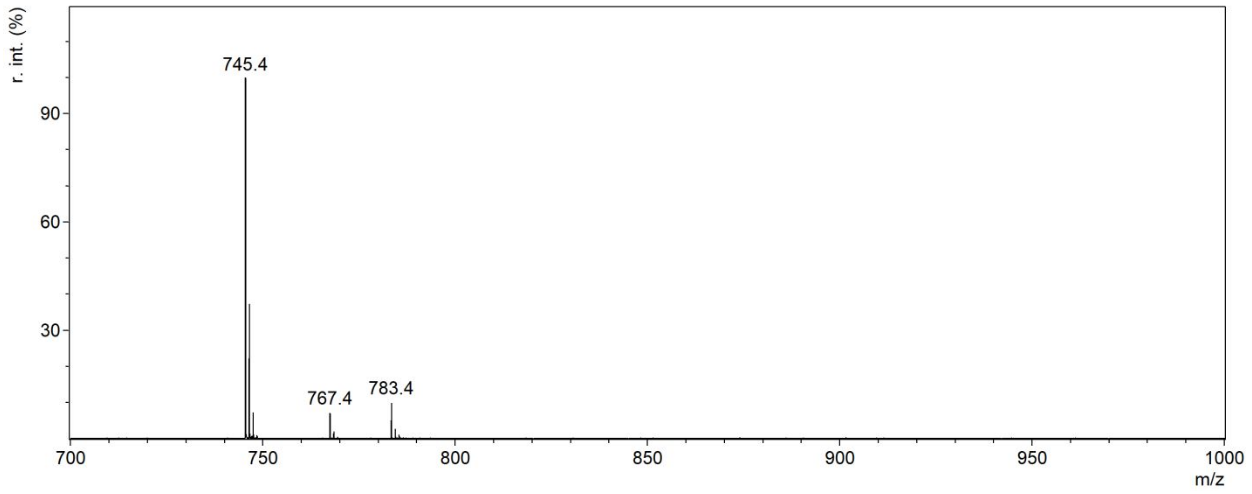

Figure S 21. MALDI-TOF spectrum of purified PDDP-Y5A (H-SQTLAAR-NH_2) in the range of m/z = 700-1000. $[M+H]^+$ = 745.4, $[M+Na]^+$ = 767.4, $[M+K]^+$ = 783.4.

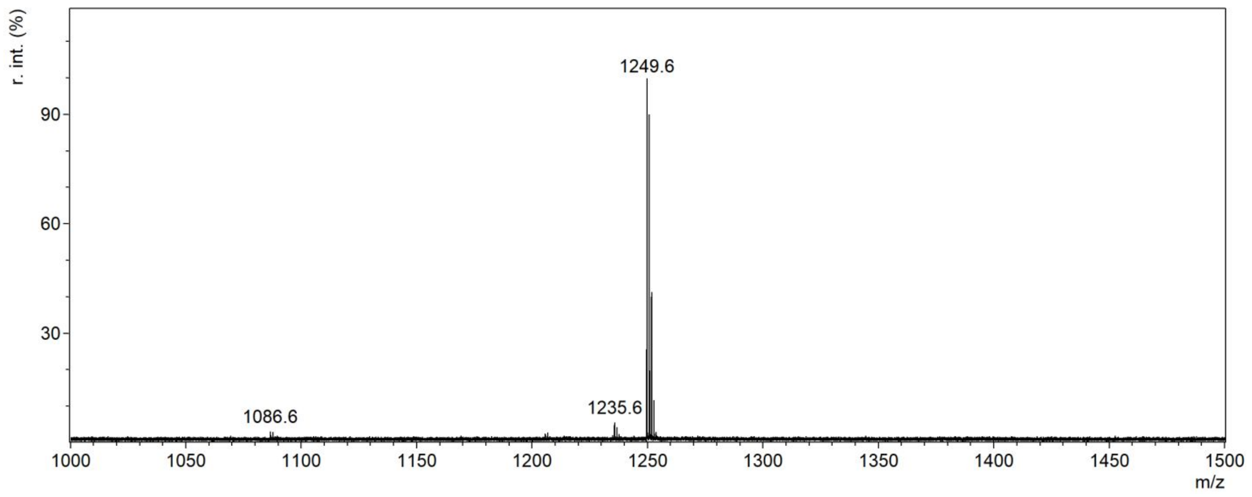

Figure S 22. MALDI-TOF spectrum of purified TAMRA-PDDP (TAMRA-SQTLYAR-NH_2) in the range of m/z = 1000-1500. $[M+H]^+$ = 1249.6.

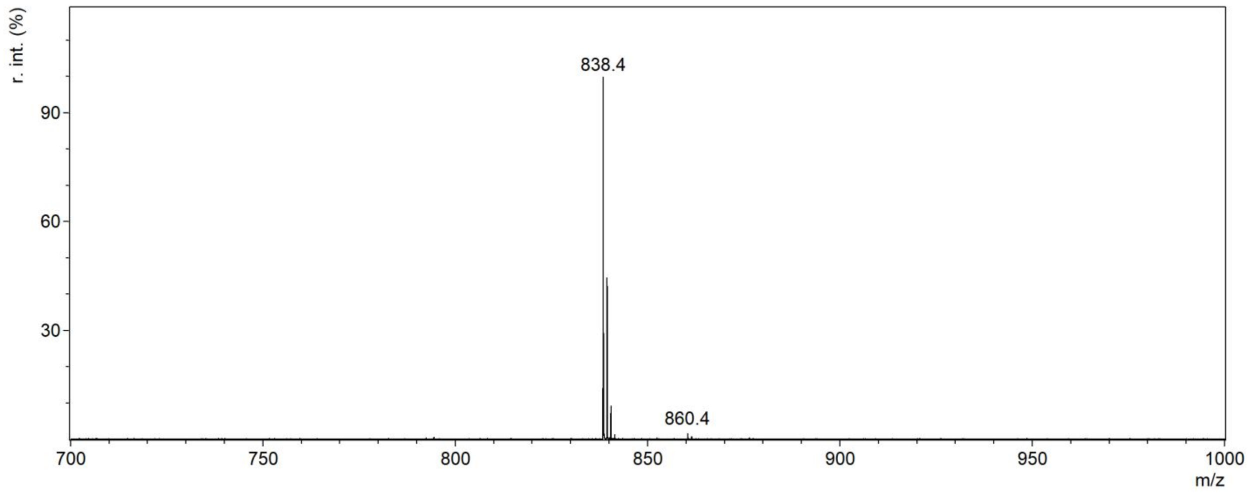

Figure S 23. MALDI-TOF spectrum of purified PDDP-OH (H-SQTLYAR-OH) in the range of m/z = 700-1000. $[M+H]^+$ = 838.4, $[M+Na]^+$ = 860.4.

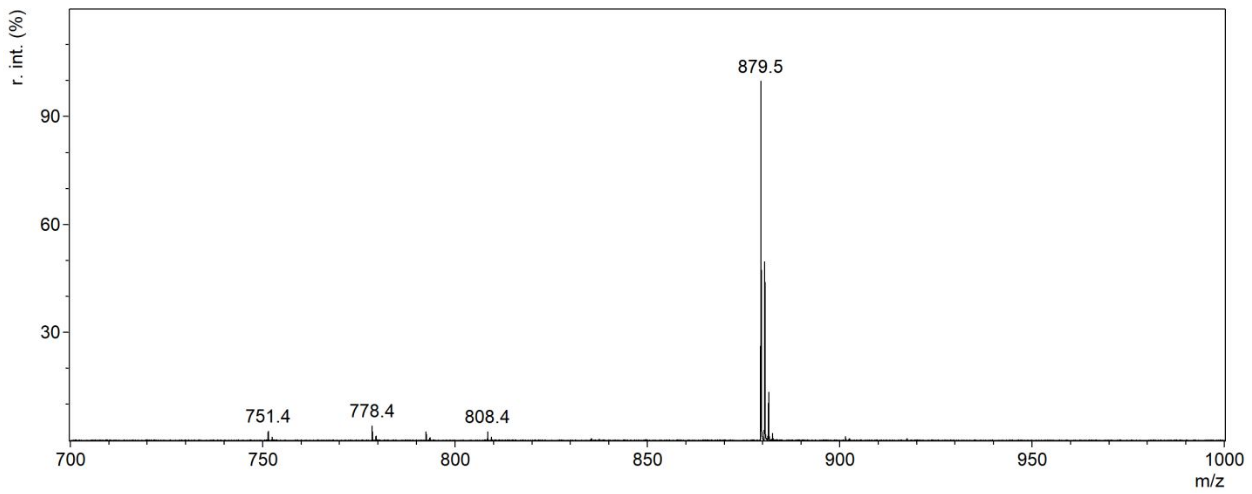

Figure S 24. MALDI-TOF spectrum of purified Ac-PDDP (Ac-SQTLYAR-NH_2) in the range of m/z = 700-1000. $[M+H]^+$ = 879.5.

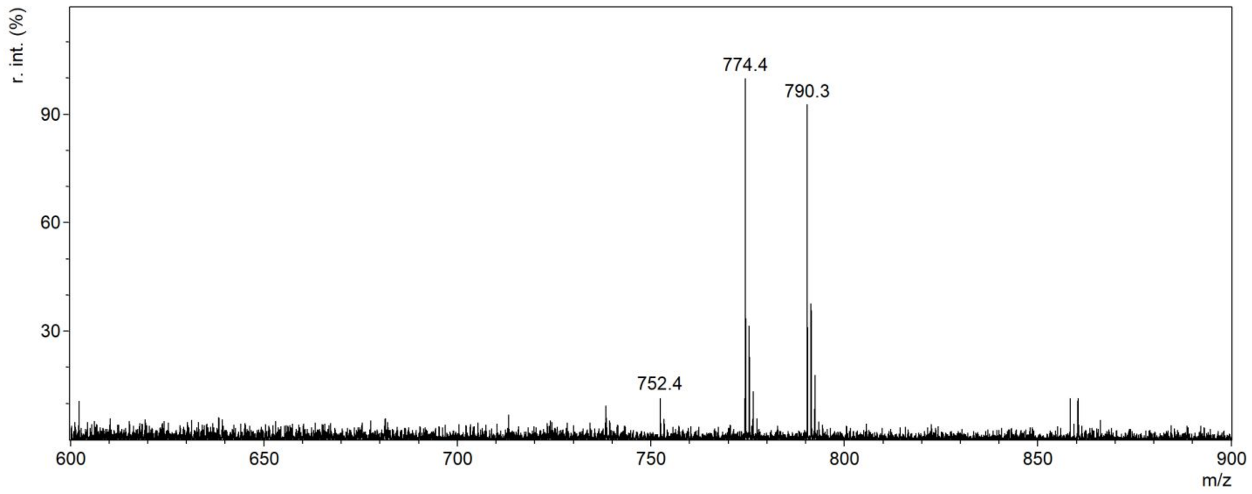

Figure S 25. MALDI-TOF spectrum of purified PDDP-R7A (H-SQTLYAA-NH_2) in the range of m/z = 600-900. $[M+H]^+$ = 752.4, $[M+Na]^+$ = 774.4, $[M+K]^+$ = 790.3.

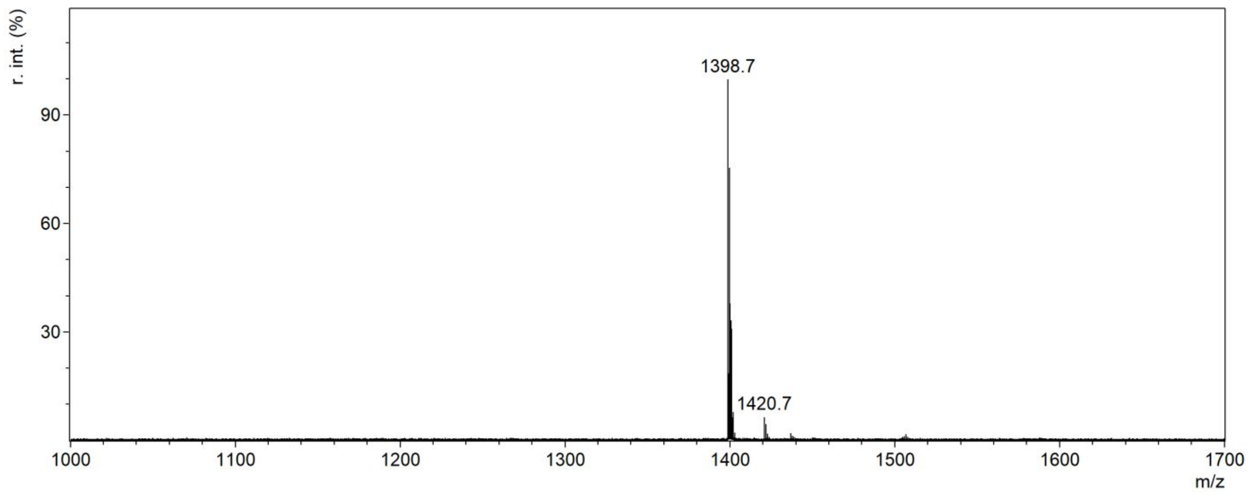

Figure S 26. MALDI-TOF spectrum of purified CelBP (H-GQVLNPYYSQCK-NH_2) in the range of m/z = 1000-1700. $[M+H]^+$ = 1398.7, $[M+Na]^+$ = 1420.7.

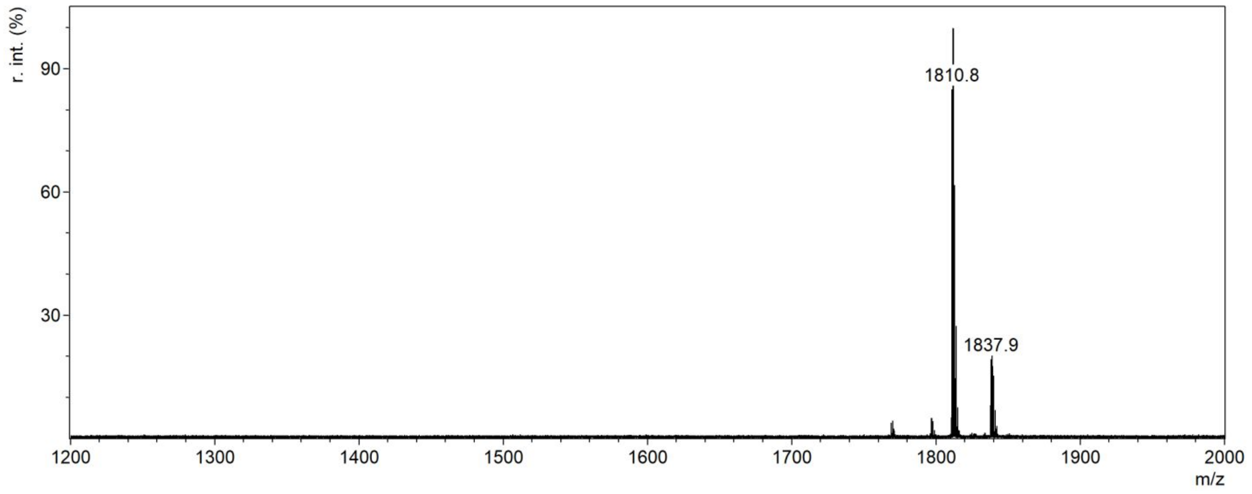

Figure S 27. MALDI-TOF spectrum of purified CelBP-TAMRA (H-GQVLNPYYSQCK(TAMRA)-NH_2) in the range of m/z = 1200-2000. $[M+H]^+$ = 1810.8.

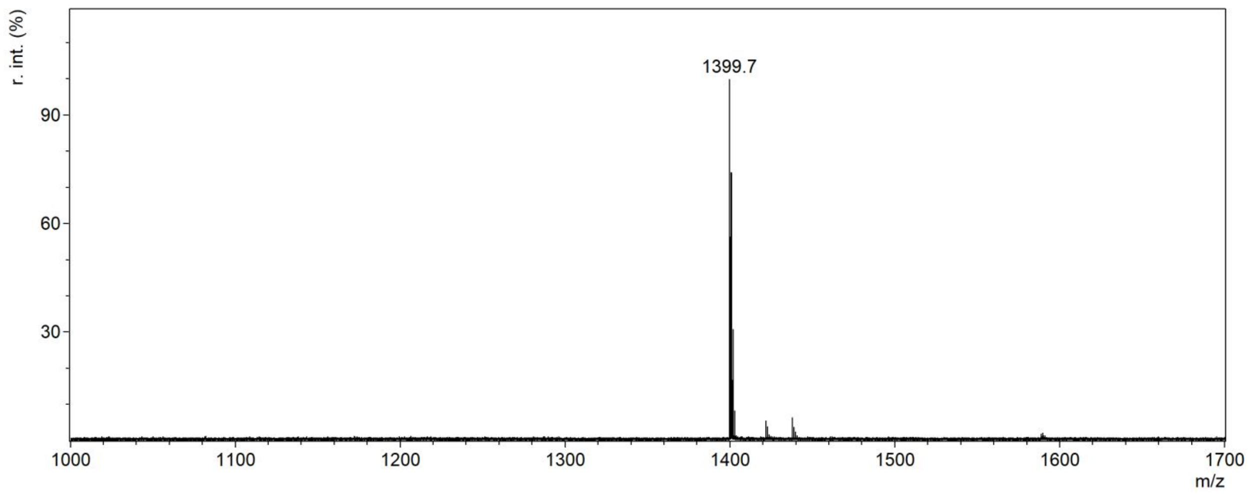

Figure S 28. MALDI-TOF spectrum of purified CelBP-N5D (H-GQVLDPYYSQCK-NH_2) in the range of m/z = 1000-1700. $[M+H]^+$ = 1399.7.

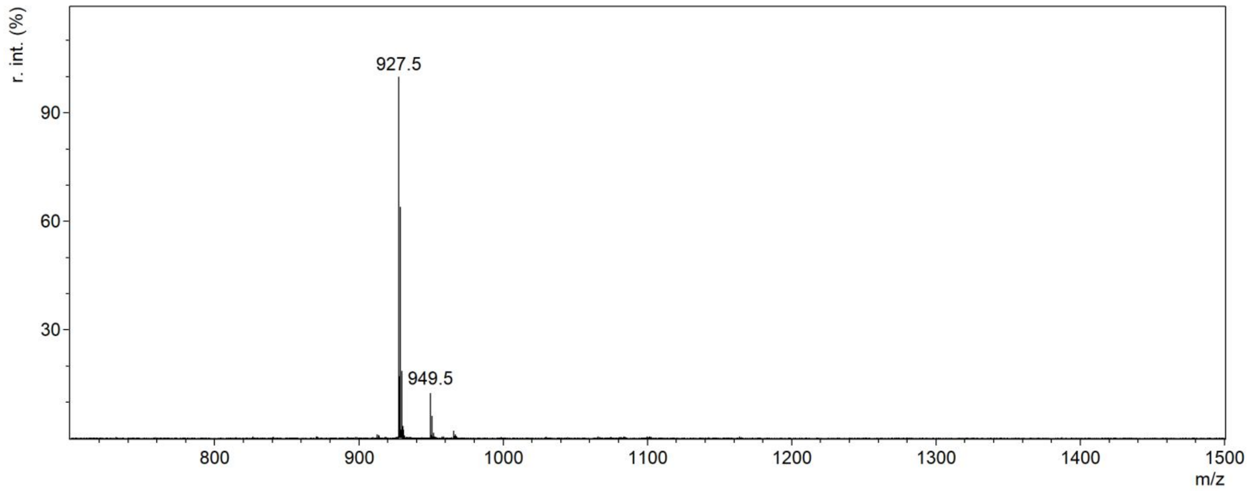

Figure S 29. MALDI-TOF spectrum of purified SM1 (H-GSITQGIPR-NH_2) in the range of m/z = 700-1500. $[M+H]^+$ = 927.5, $[M+Na]^+$ = 949.5.

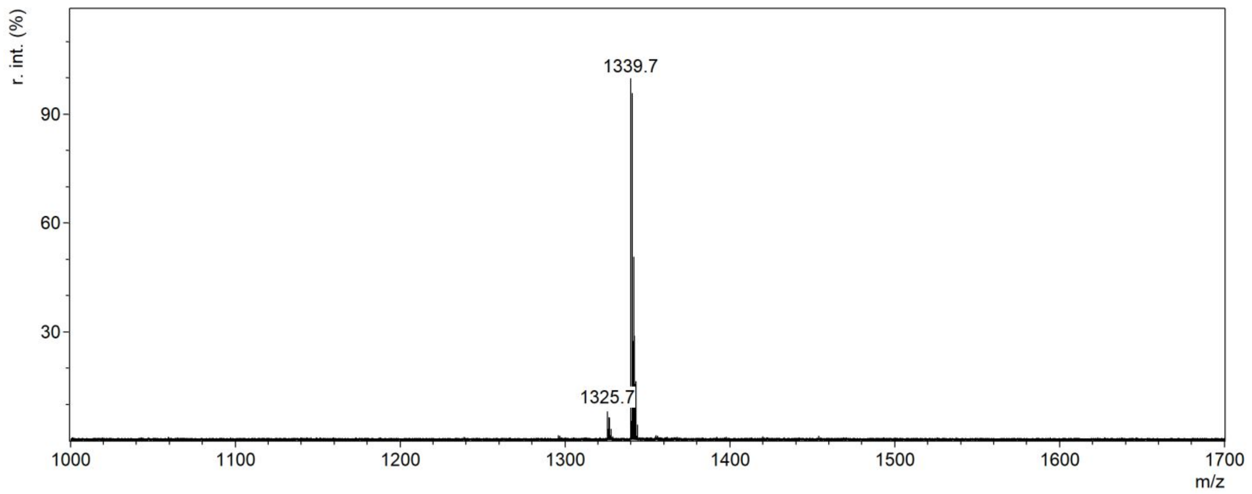

Figure S 30. MALDI-TOF spectrum of purified TAMRA-SM1 (TAMRA-GSITQGIPR-NH_2) in the range of m/z = 1000-1700. $[M+H]^+$ = 1339.7.

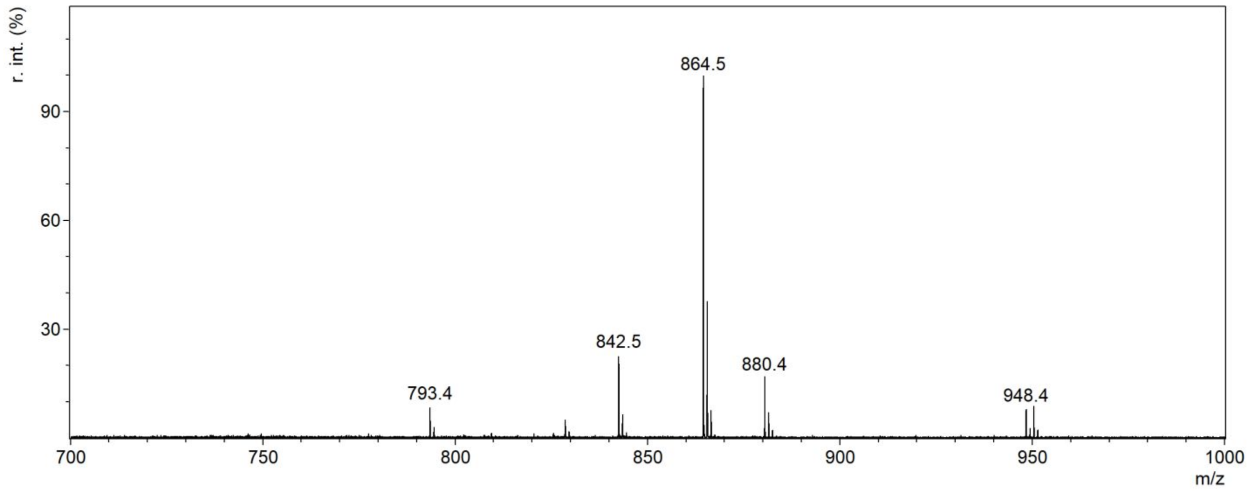

Figure S 31. MALDI-TOF spectrum of purified SM1-R9A (H-GSITQGIPA-NH_2) in the range of m/z = 700-1000. $[M+H]^+$ = 842.5, $[M+Na]^+$ = 864.5, $[M+K]^+$ = 880.4.

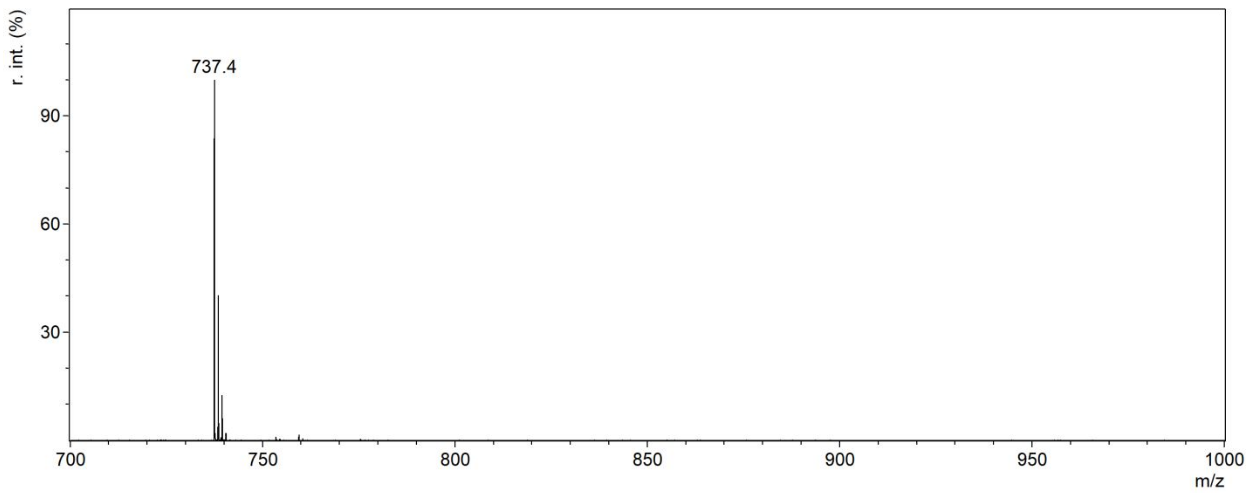

Figure S 32. MALDI-TOF spectrum of purified AAP (H-RAYVVM-NH_2) in the range of m/z = 700-1000. $[M+H]^+$ = 737.4.

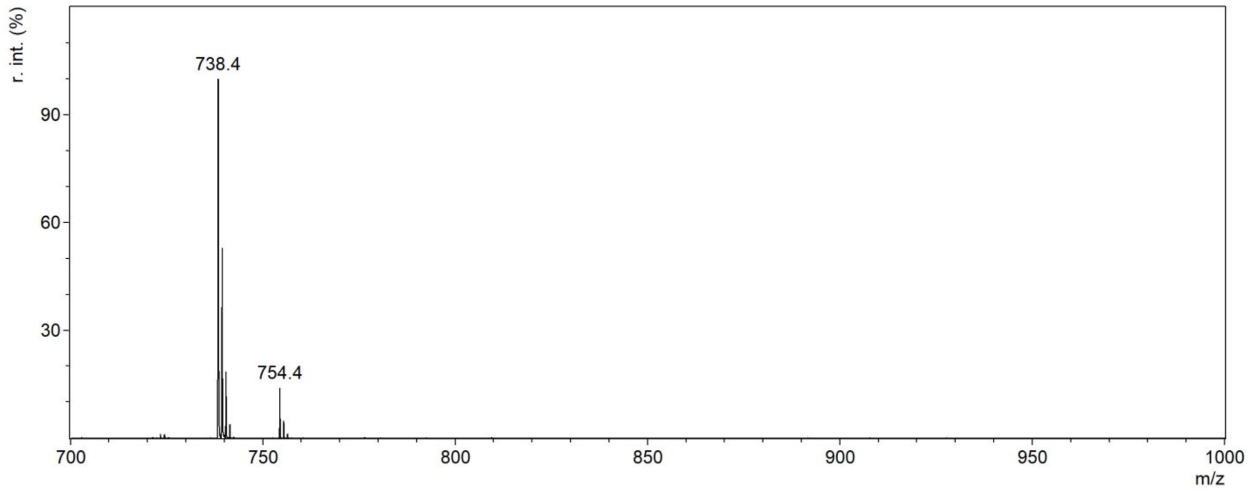

Figure S 33. MALDI-TOF spectrum of purified AAP-OH (H-RAYVVM-OH) in the range of m/z = 700-1000. $[M+H]^+$ = 738.4, $[M+Na]^+$ = 754.4.

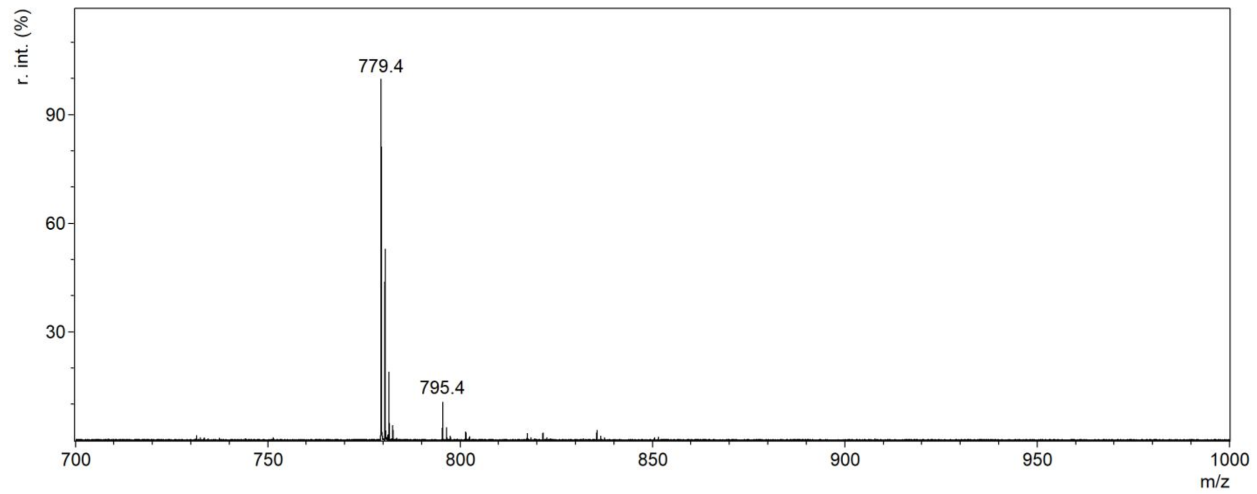

Figure S 34. MALDI-TOF spectrum of purified Ac-AAP (Ac-RAYVVM-NH_2) in the range of m/z = 700-1000. $[M+H]^+$ = 779.4.

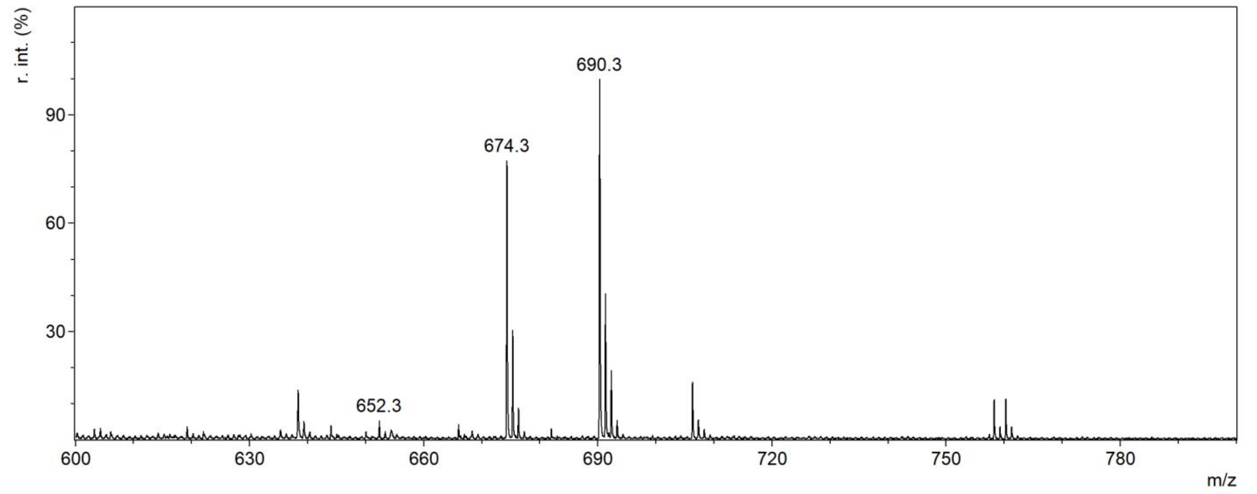

Figure S 35. MALDI-TOF spectrum of purified AAP-R1A (H-AAYVVM-NH_2) in the range of m/z = 600-800. $[M+H]^+$ = 652.3, $[M+Na]^+$ = 674.3, $[M+H]^+$ = $[M+K]^+$ = 690.3.

3. HPLC chromatograms of the purified peptides

The two regioisomers 5- and 6-TAMRA were used in the synthesis of the labeled peptides. This results in two peaks in the corresponding purity chromatograms.

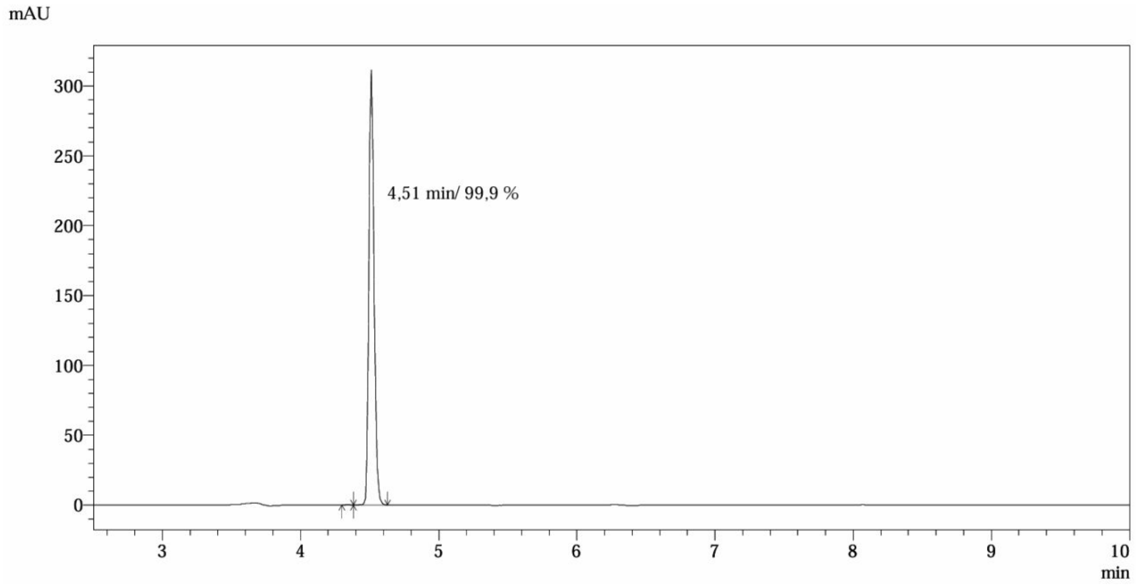

Figure S 36. Chromatogram of the HPLC analysis of PDDP (H-SQTLYAR-NH_2) (ß = 200 µg/mL). Elution conditions according to the analytical method described in the paper. Y-axis = absorbance at 220 nm.

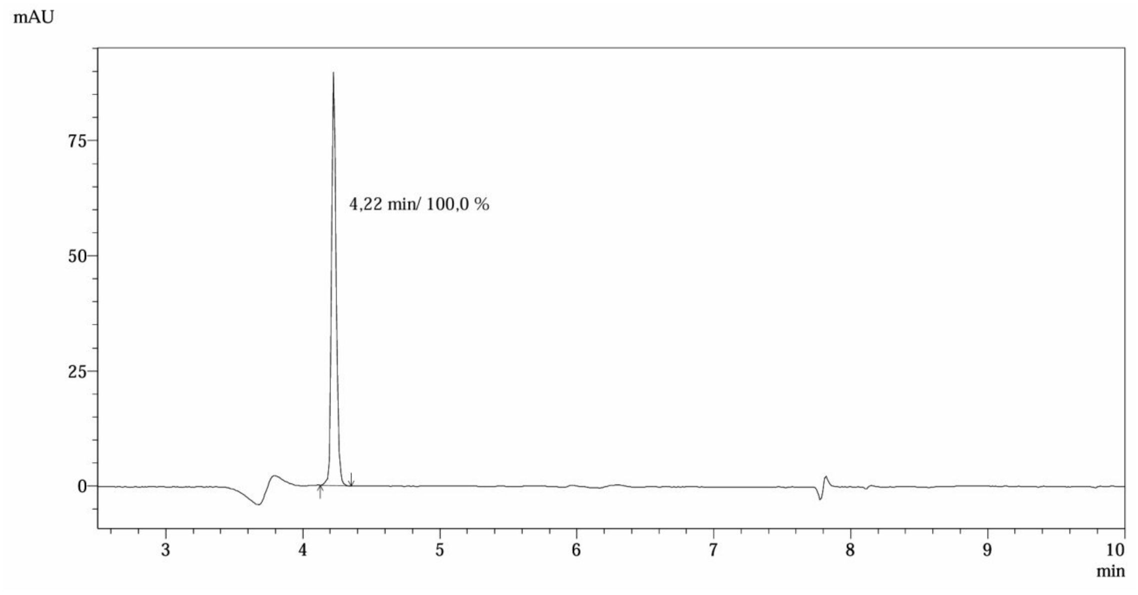

Figure S 37. Chromatogram of the HPLC analysis of PDDP-Y5S (H-SQTLSAR-NH_2 (ß = 200 µg/mL). Elution conditions according to the analytical method described in the paper. Y-axis = absorbance at 220 nm.

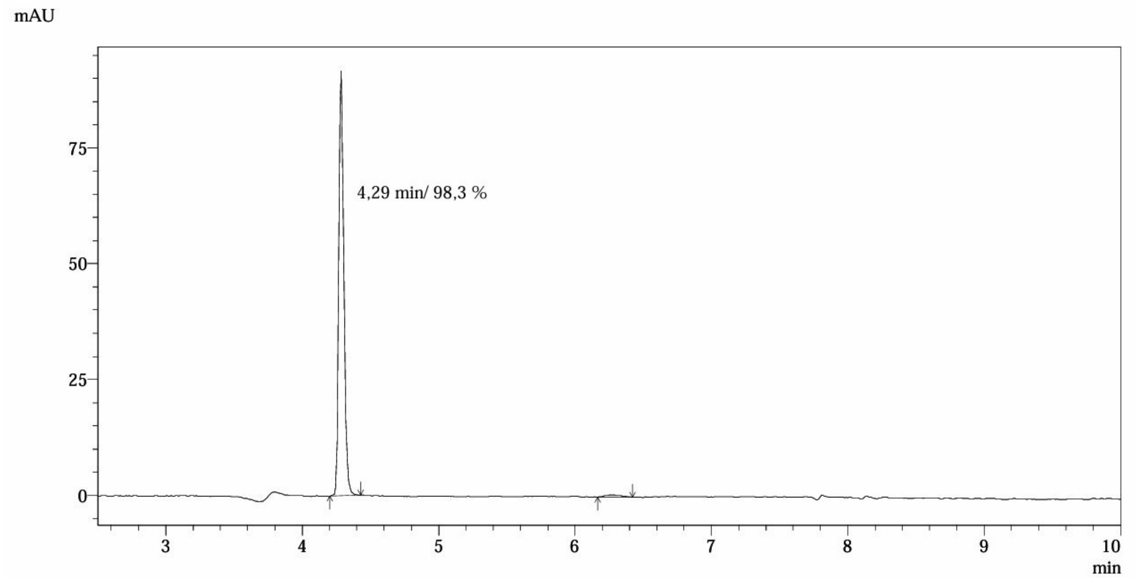

Figure S 38. Chromatogram of the HPLC analysis of PDDP-Y5A (H-SQTLAAR-NH_2) (ß = 200 µg/mL). Elution conditions according to the analytical method described in the paper. Y-axis = absorbance at 220 nm.

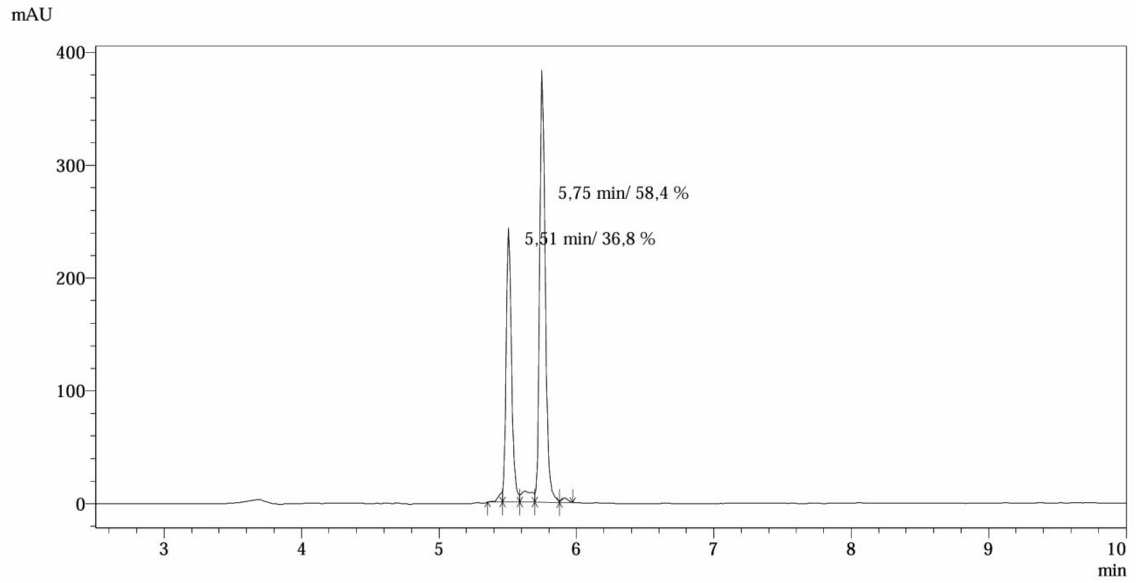

Figure S 39. Chromatogram of the HPLC analysis of TAMRA-PDDP (TAMRA-SQTLYAR-NH_2) (ß = 200 µg/mL). Elution conditions according to the analytical method described in the paper. Y-axis = absorbance at 220 nm.

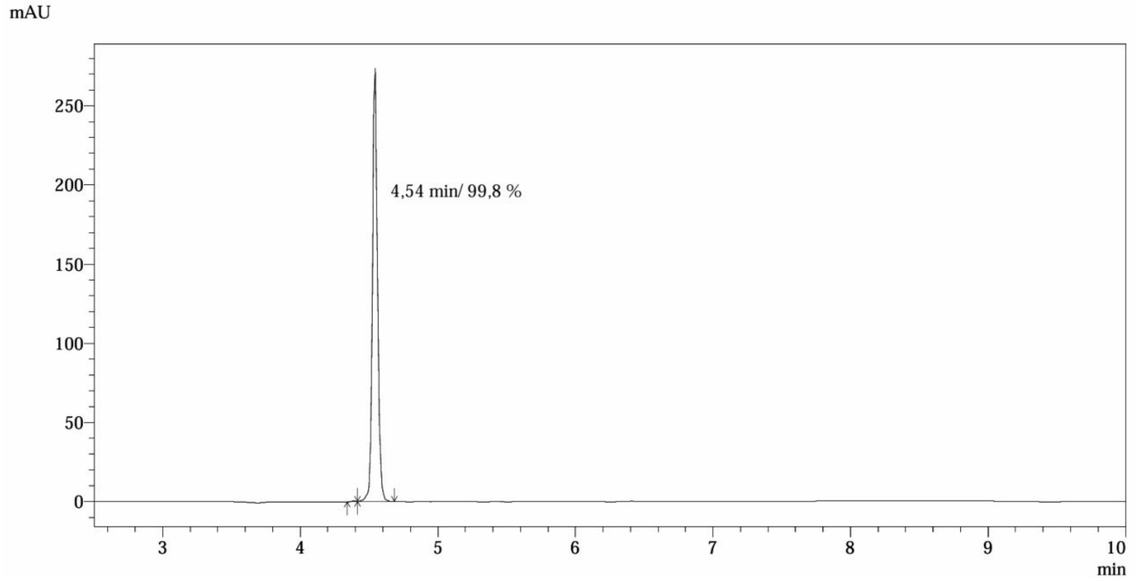

Figure S 40. Chromatogram of the HPLC analysis of PDDP-OH (H-SQTLYAR-OH) (ß = 200 µg/mL). Elution conditions according to the analytical method described in the paper. Y-axis = absorbance at 220 nm.

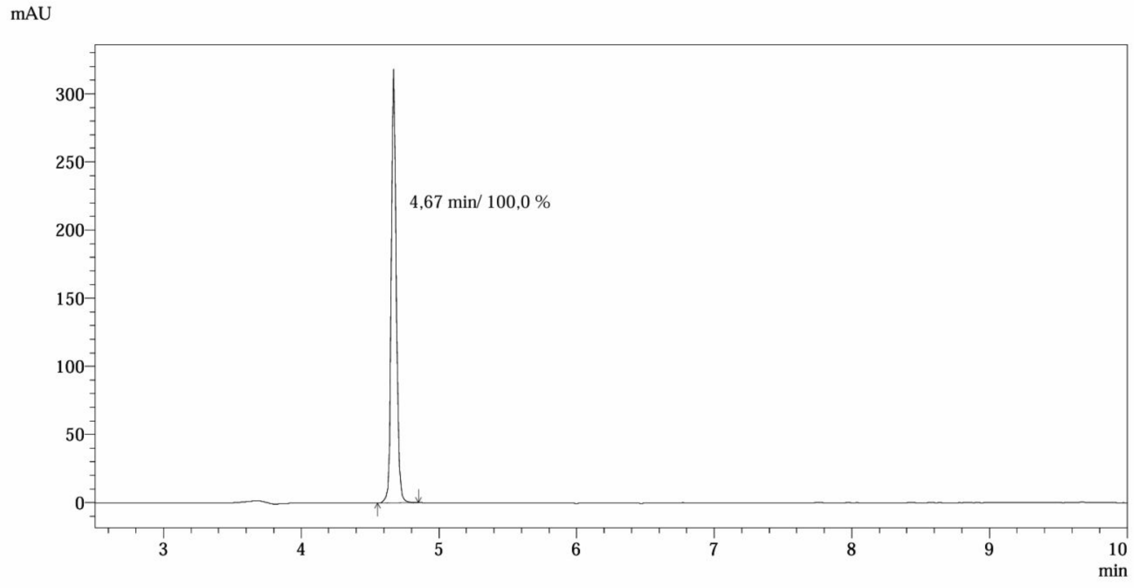

Figure S 41. Chromatogram of the HPLC analysis of PDDP-R7A (H-SQTLYAA-NH_2) (ß = 200 µg/mL). Elution conditions according to the analytical method described in the paper. Y-axis = absorbance at 220 nm.

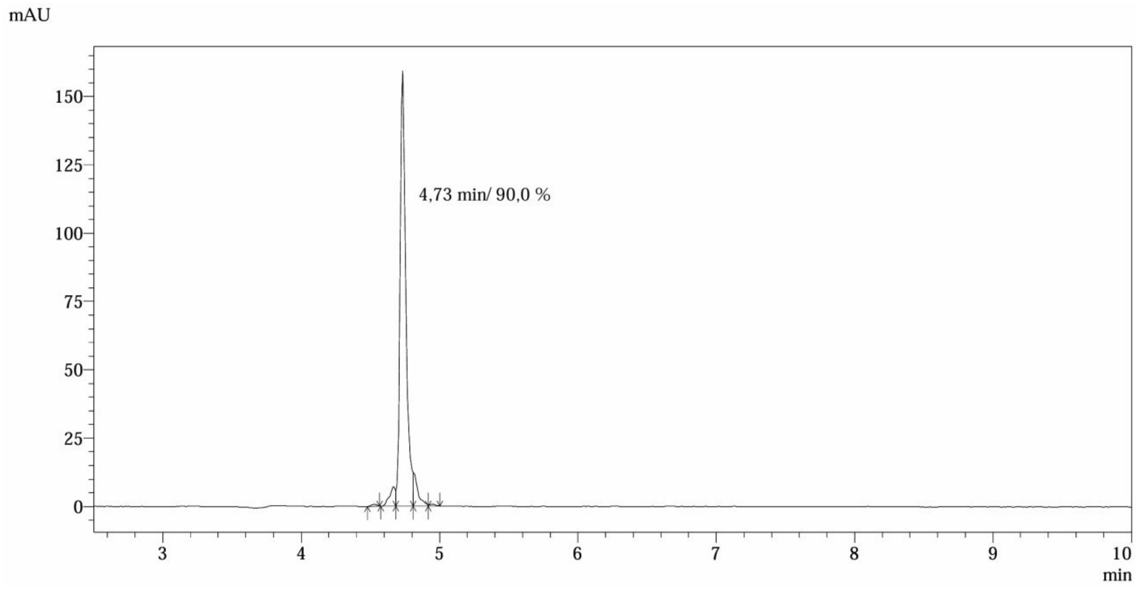

Figure S 42. Chromatogram of the HPLC analysis of Ac-PDDP (Ac-SQTLYAR-NH_2) (ß = 200 µg/mL). Elution conditions according to the analytical method described in the paper. Y-axis = absorbance at 220 nm.

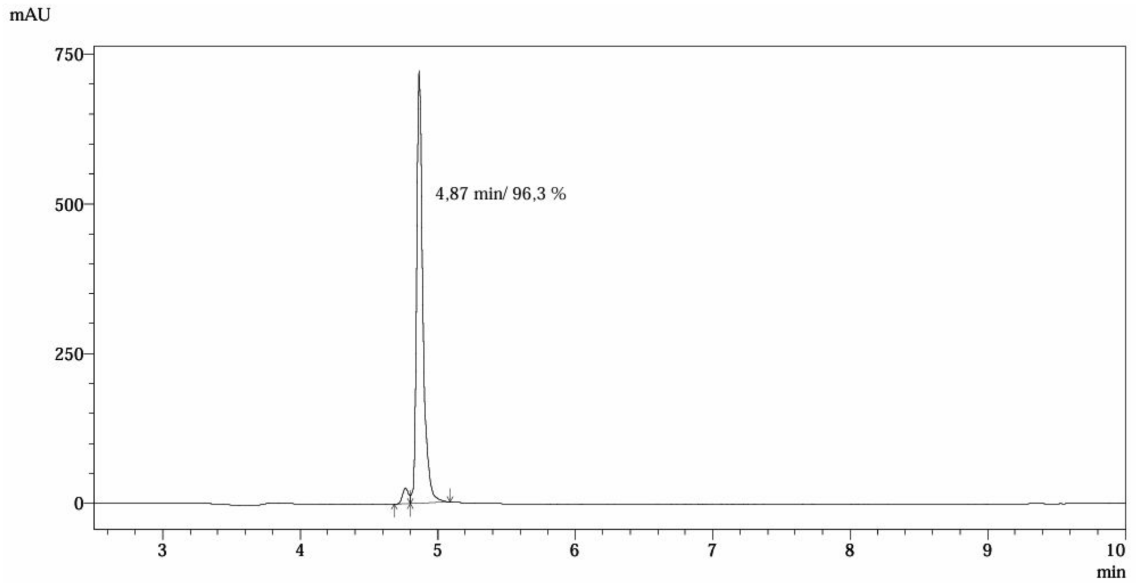

Figure S 43. Chromatogram of the HPLC analysis of CelBP (H-GQVLNPYYSQCK-NH_2) (ß = 200 µg/mL). Elution conditions according to the analytical method described in the paper. Y-axis = absorbance at 220 nm.

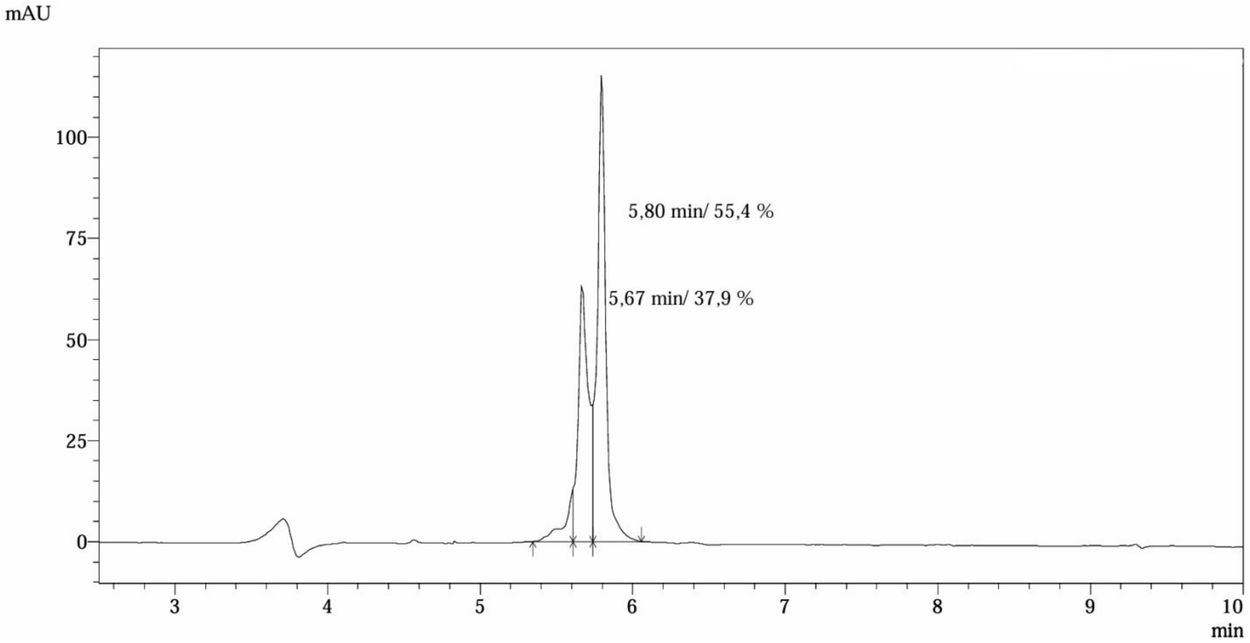

Figure S 44. Chromatogram of the HPLC analysis of CelBP-TAMRA (H-GQVLNPYYSQCK(TAMRA)-NH_2) (ß = 200 µg/mL). Elution conditions according to the analytical method described in the paper. Y-axis = absorbance at 220 nm.

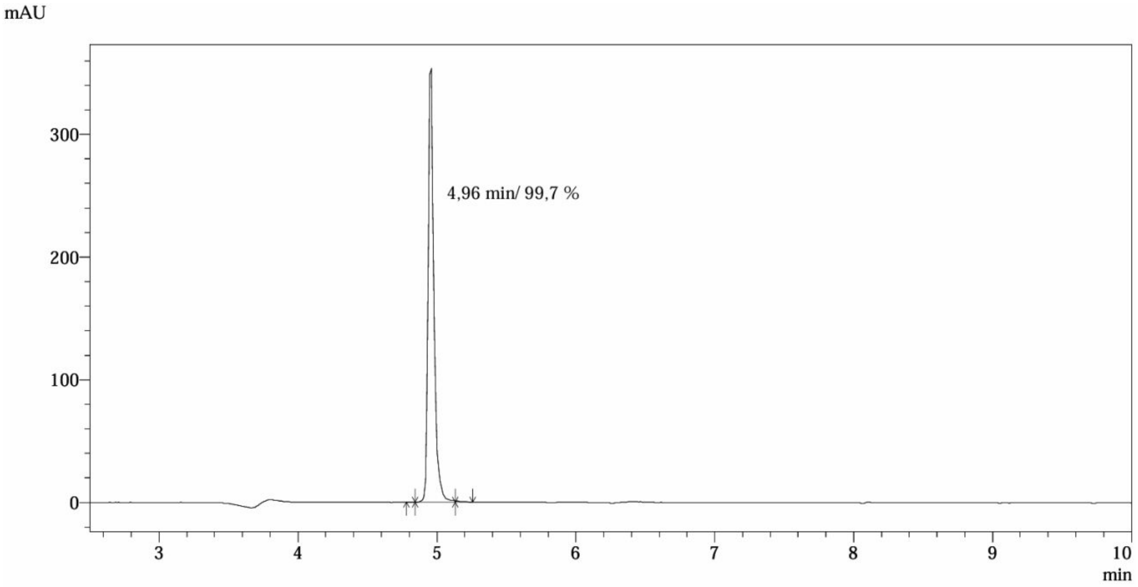

Figure S 45. Chromatogram of the HPLC analysis of CelBP-N5D (H-GQVLDPYYSQCK-NH_2) (ß = 200 µg/mL). Elution conditions according to the analytical method described in the paper. Y-axis = absorbance at 220 nm.

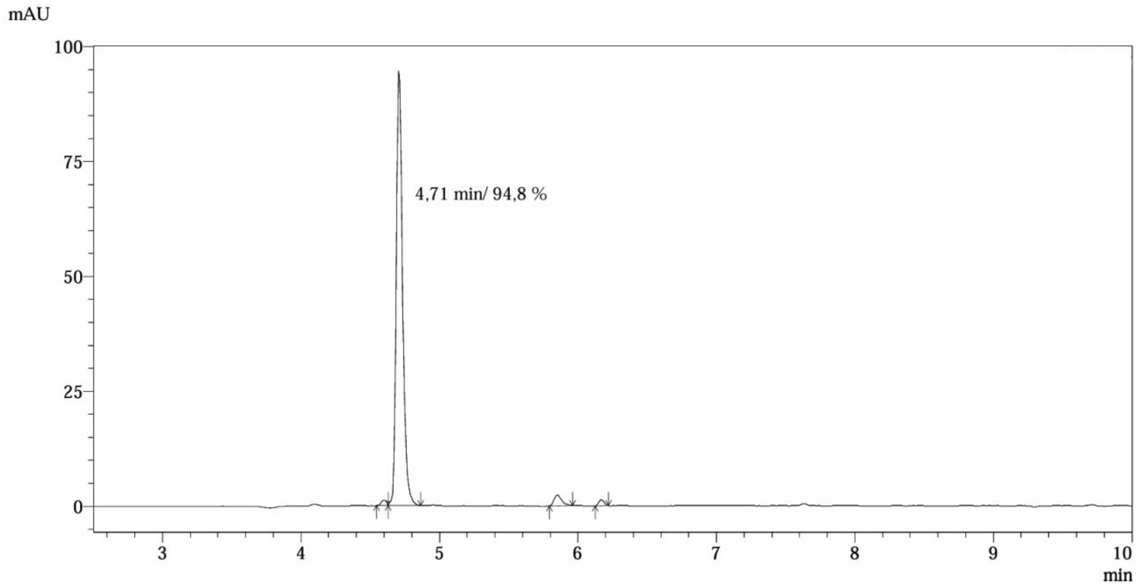

Figure S 46. Chromatogram of the HPLC analysis of SM1 (H-GSITQGIPA-NH_2) (ß = 200 µg/mL). Elution conditions according to the analytical method described in the paper. Y-axis = absorbance at 220 nm.

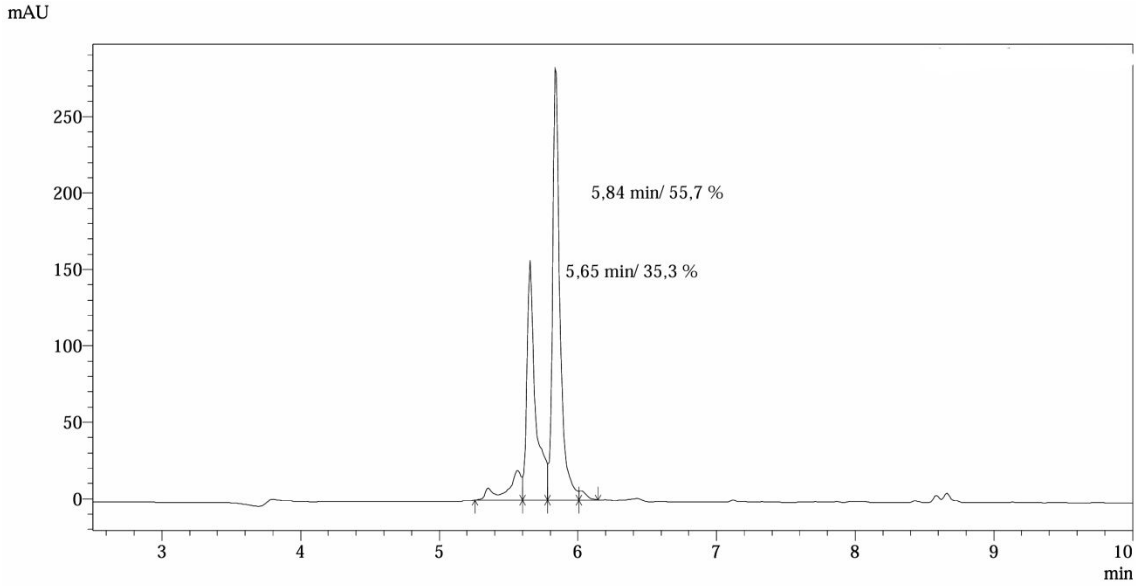

Figure S 47. Chromatogram of the HPLC analysis of TAMRA-SM1 (TAMRA-GSITQGIPA-NH_2) (ß = 200 µg/mL). Elution conditions according to the analytical method described in the paper. Y-axis = absorbance at 220 nm.

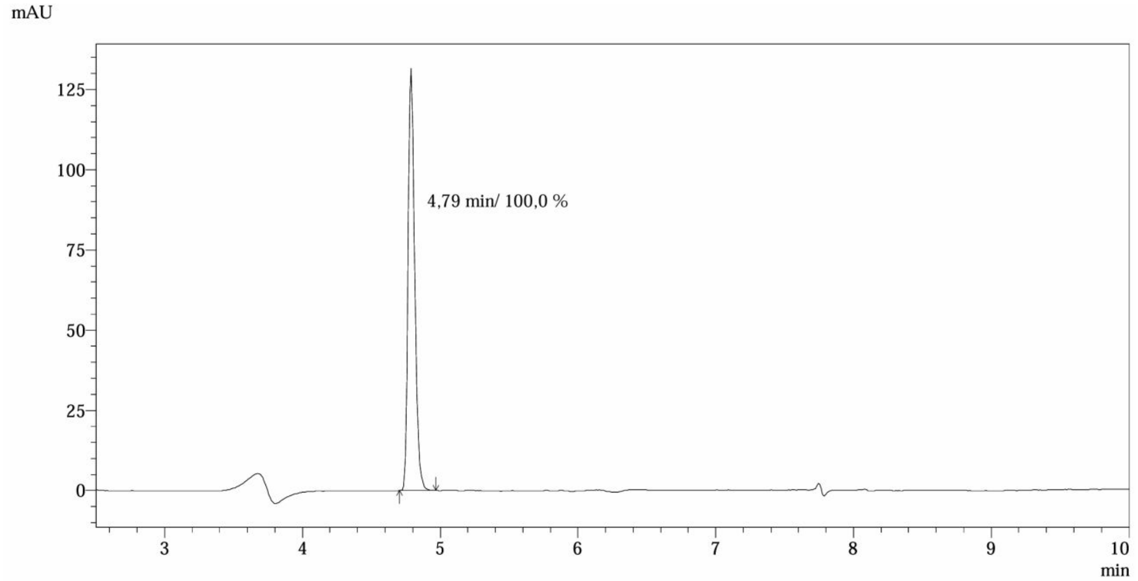

Figure S 48. Chromatogram of the HPLC analysis of SM1-R9A (H-GSITQGIPA-NH_2) (ß = 200 µg/mL). Elution conditions according to the analytical method described in the paper. Y-axis = absorbance at 220 nm.

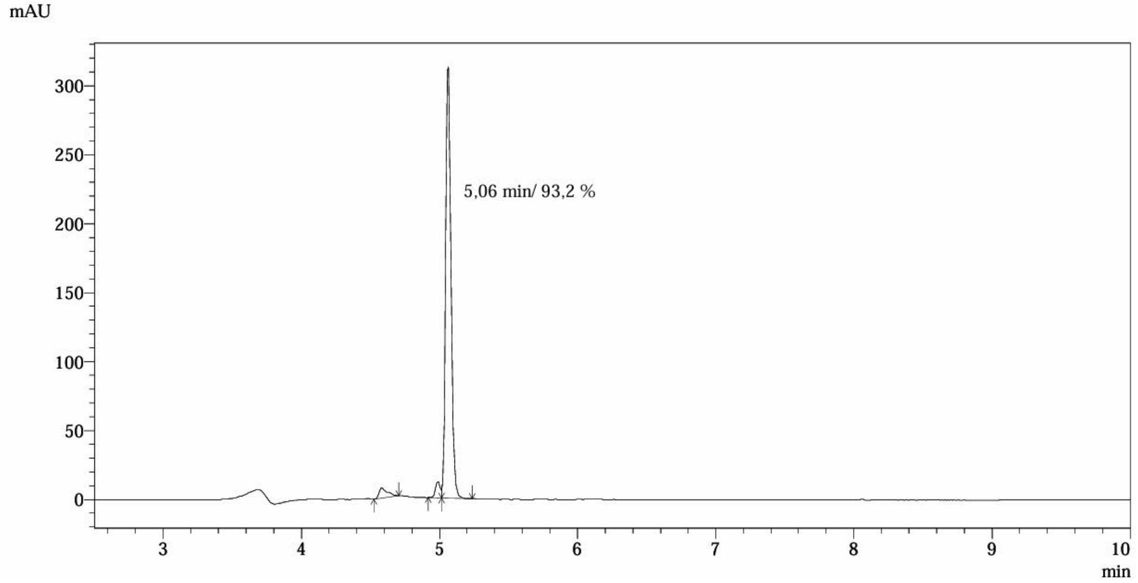

Figure S 49. Chromatogram of the HPLC analysis of AAP (H-RAYVVM-NH_2) (ß = 200 µg/mL). Elution conditions according to the analytical method described in the paper. Y-axis = absorbance at 220 nm.

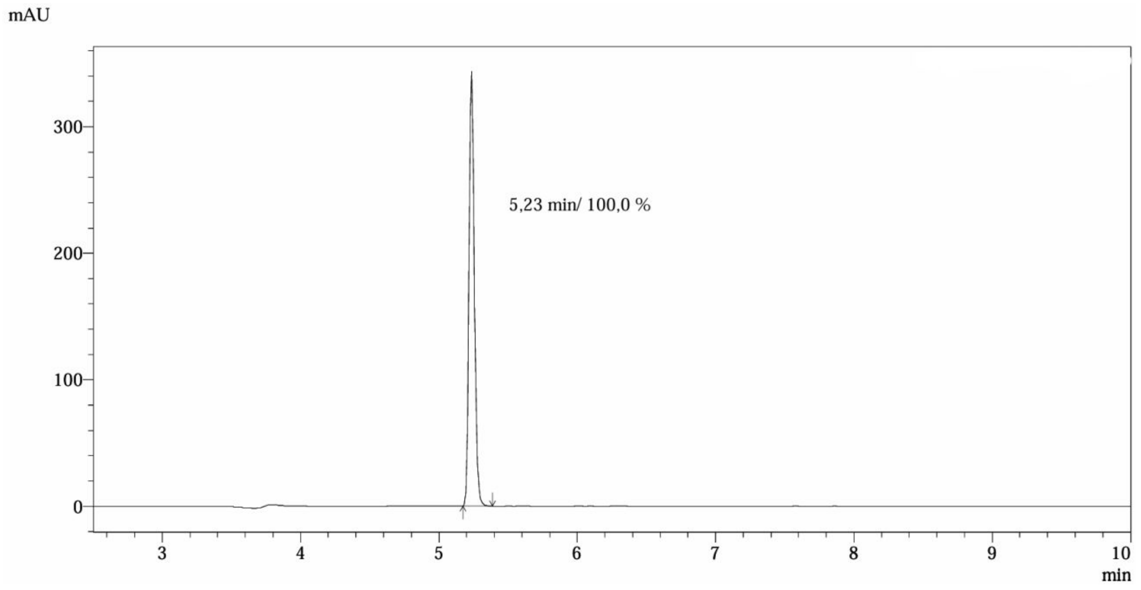

Figure S 50. Chromatogram of the HPLC analysis of AAP-OH (H-RAYVVM-OH) (ß = 200 µg/mL). Elution conditions according to the analytical method described in the paper. Y-axis = absorbance at 220 nm.

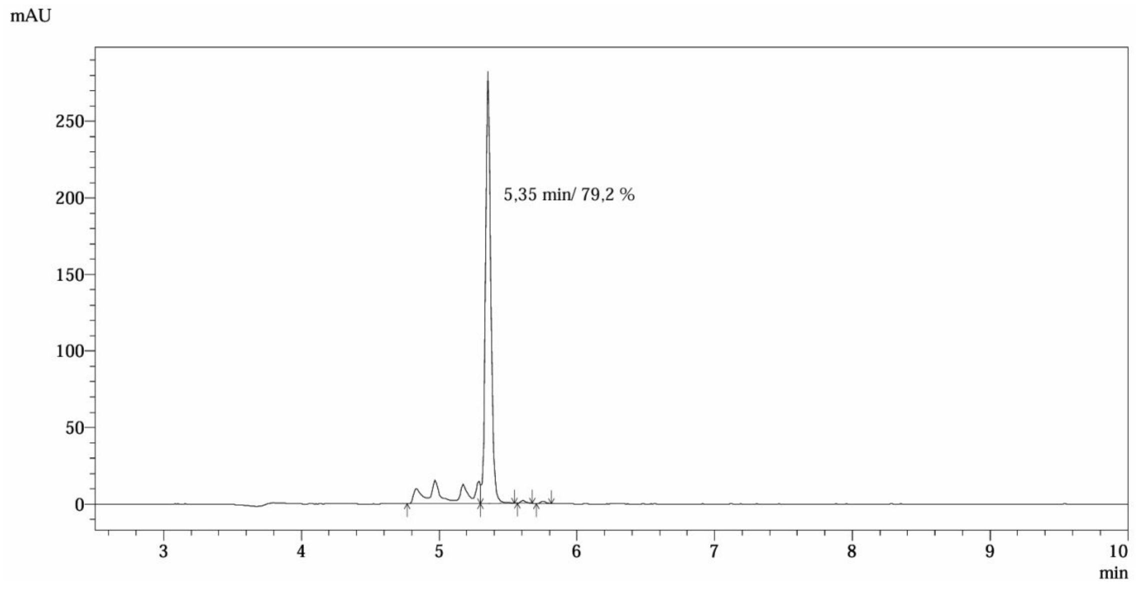

Figure S 51. Chromatogram of the HPLC analysis of Ac-AAP (Ac-RAYVVM-NH_2) (ß = 200 µg/mL). Elution conditions according to the analytical method described in the paper. Y-axis = absorbance at 220 nm.

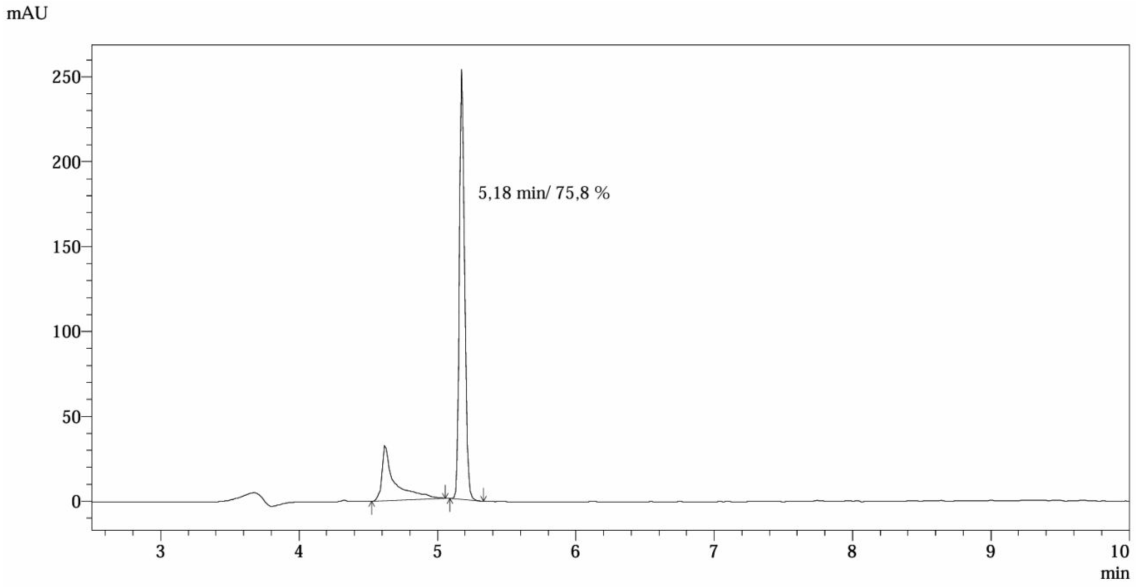

Figure S 52. Chromatogram of the HPLC analysis of AAP-R1A (H-AAYVVM-NH_2) (ß = 200 µg/mL). Elution conditions according to the analytical method described in the paper. Y-axis = absorbance at 220 nm.

4. UV/Vis spectra of the purified peptides

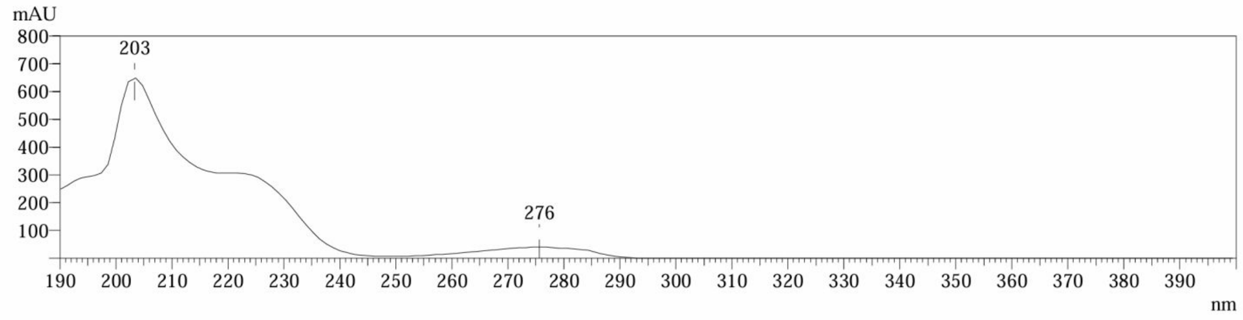

Figure S 53. Absorbance spectrum of the peak of PDDP (H-SQTLYAR-NH_2) from 190 nm – 400 nm. Use of the HPLC analytical method described in the paper. Injection volume = 10 µL, ß = 200 µg/mL.

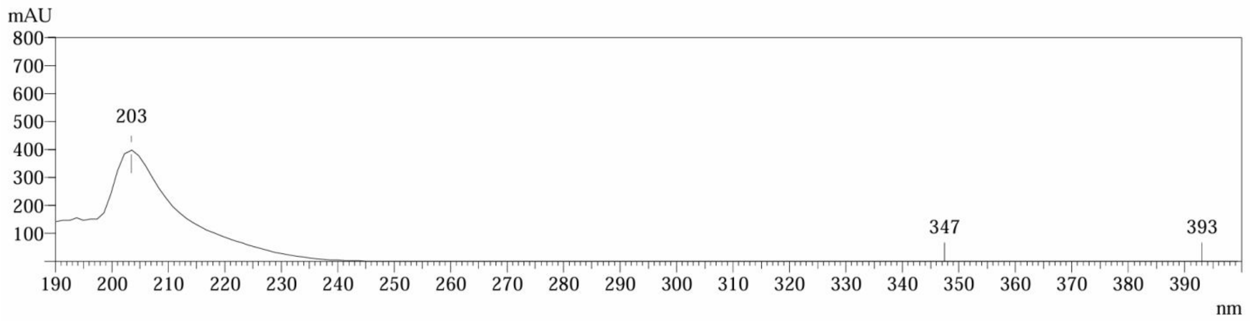

Figure S 54. Absorbance spectrum of the peak of PDDP-Y5S (H-SQTLSAR-NH_2) from 190 nm - 400 nm. Use of the HPLC analytical method described in the paper. Injection volume = 10 µL, ß = 200 µg/mL.

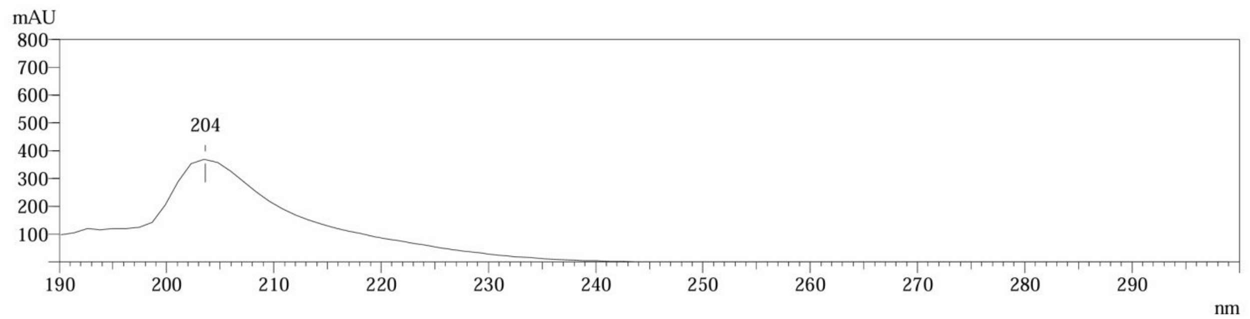

Figure S 55. Absorbance spectrum of the peak of PDDP-Y5A (H-SQTLAAR-NH_2) from 190 nm - 300 nm. Use of the HPLC analytical method described in the paper. Injection volume = 10 µL, ß = 200 µg/mL.

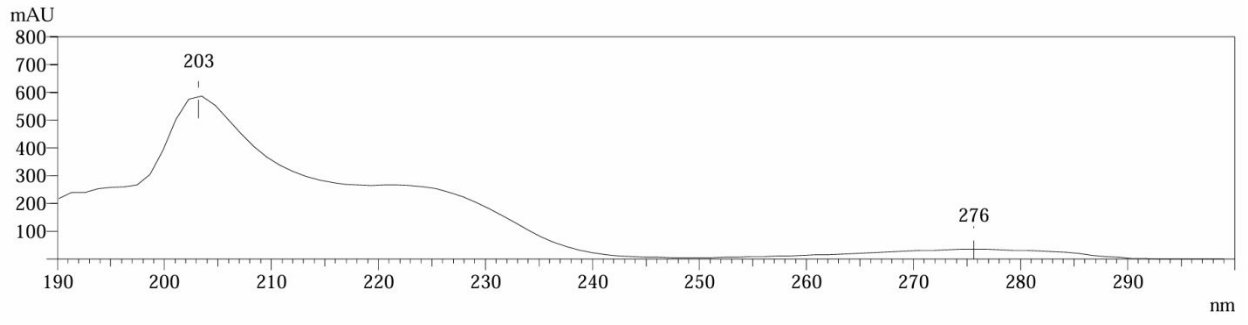

Figure S 56. Absorbance spectrum of the peak of PDDP-OH (H-SQTLYAR-OH) from 190 nm - 300 nm. Use of the HPLC analytical method described in the paper. Injection volume = 10 µL, ß = 200 µg/mL.

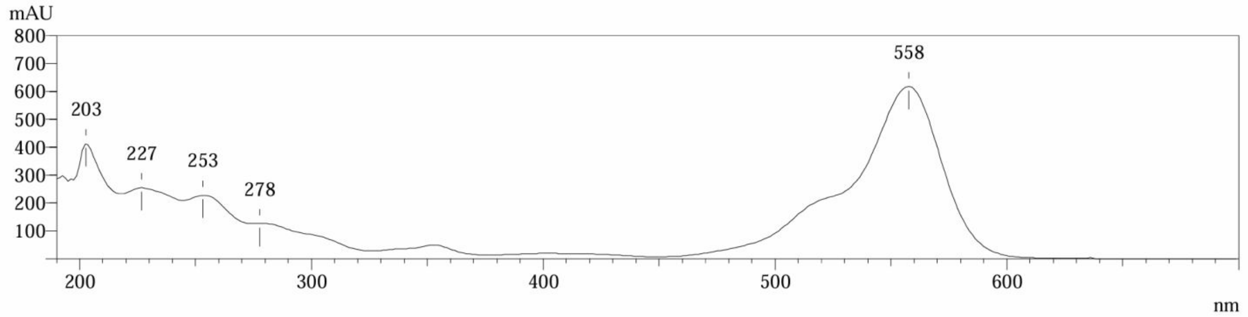

Figure S 57. Absorbance spectrum of the peak at 5.51 min of TAMRA-PDDP (TAMRA-SQTLYAR-NH_2) from 190 nm - 700 nm. Use of the HPLC analytical method described in the paper. Injection volume = 10 µL, ß = 200 µg/mL.

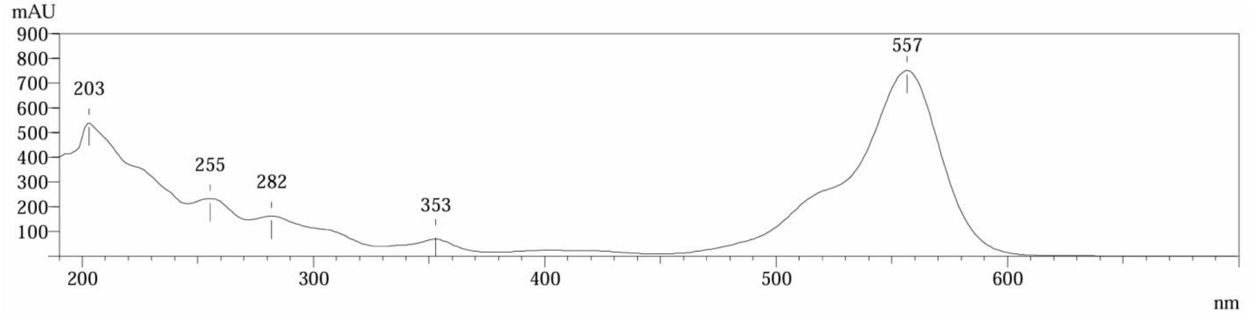

Figure S 58. Absorbance spectrum of the peak at 5.75 min of TAMRA-PDDP (TAMRA-SQTLYAR-NH_2) from 190 nm - 700 nm. Use of the HPLC analytical method described in the paper. Injection volume = 10 µL, ß = 200 µg/mL.

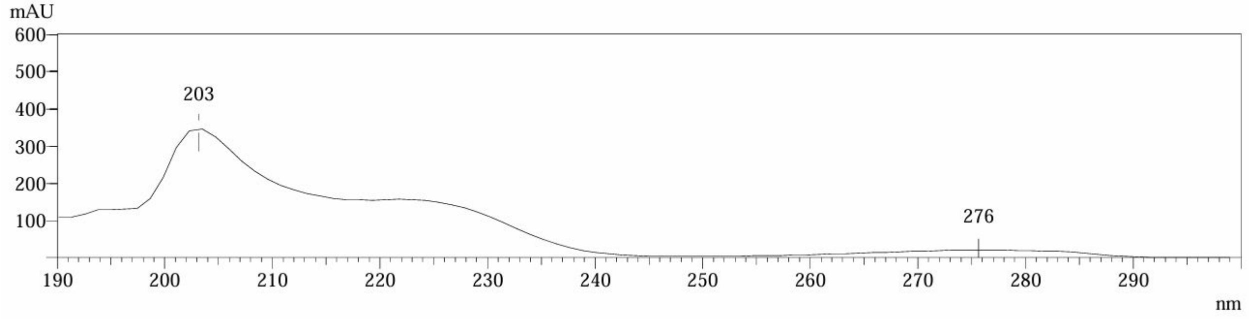

Figure S 59. Absorbance spectrum of the peak of Ac-PDDP (Ac-SQTLYAR-NH_2) from 190 nm - 300 nm. Use of the HPLC analytical method described in the paper. Injection volume = 10 µL, ß = 200 µg/mL.

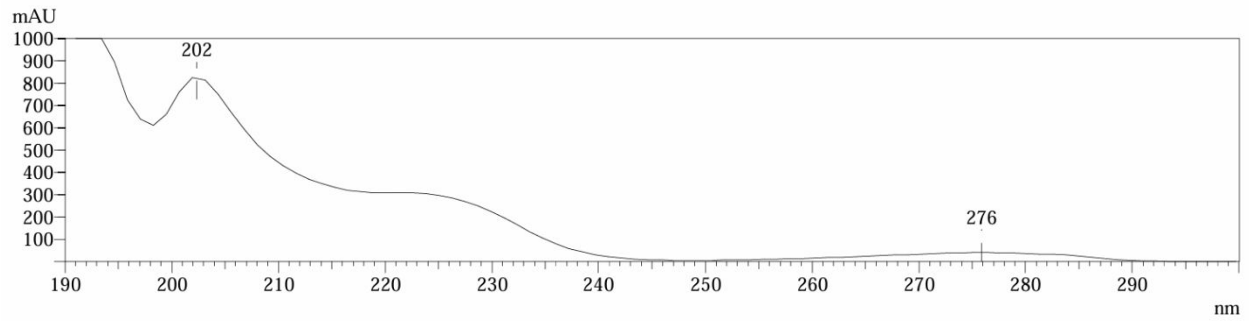

Figure S 60. Absorbance spectrum of the peak at 4.67 min of PDDP-R7A (H-SQTLYAA-NH_2) from 190 nm - 300 nm. Use of the HPLC analytical method described in the paper. Injection volume = 10 µL, ß = 200 µg/mL.

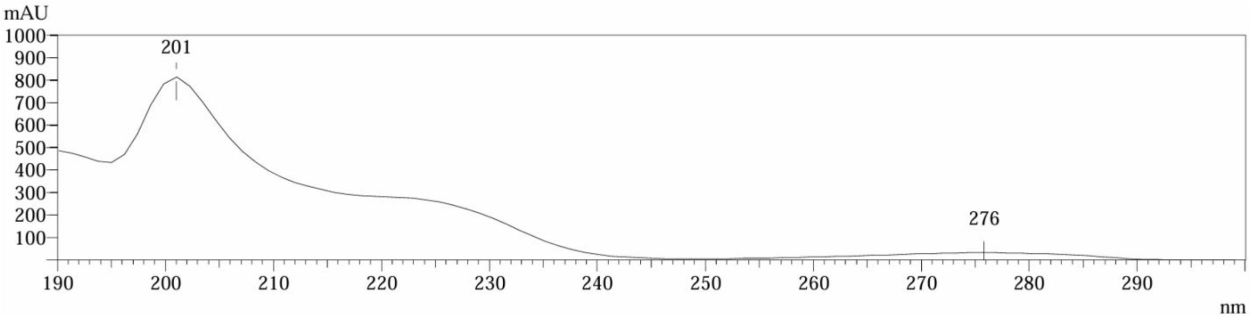

Figure S 61. Absorbance spectrum of the peak at 5.51 min of CelBP (H-GQVLNPYYSQCK-NH_2) from 190 nm - 700 nm. Use of the HPLC analytical method described in the paper. Injection volume = 10 µL, ß = 200 µg/mL.

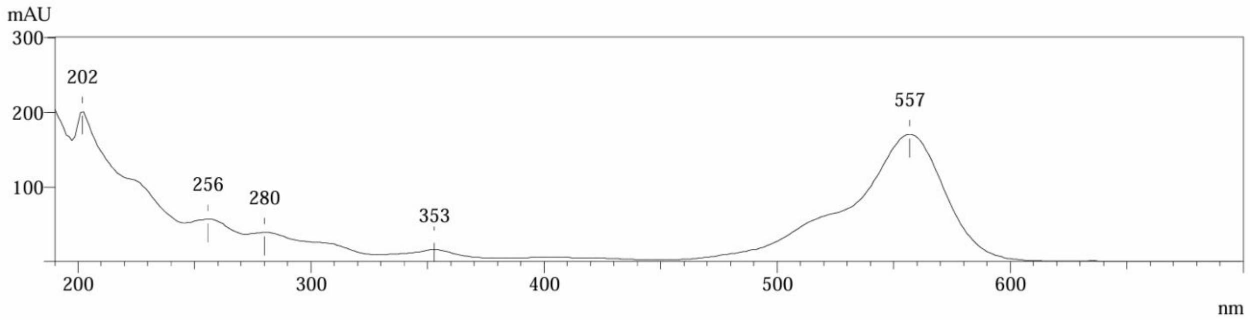

Figure S 62. Absorbance spectrum of the peak at 5.51 min of CelBP-TAMRA (H-GQVLNPYYSQCK(TAMRA)-NH_2) from 190 nm - 700 nm. Use of the HPLC analytical method described in the paper. Injection volume = 10 µL, ß = 200 µg/mL.

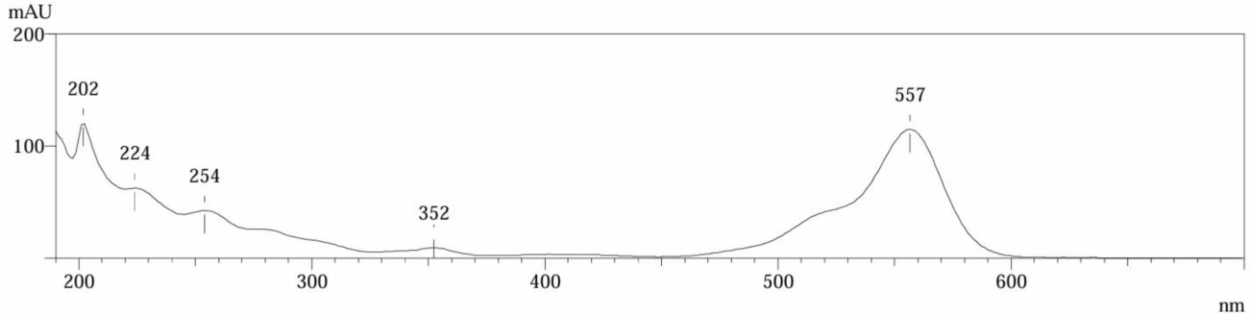

Figure S 63. Absorbance spectrum of the peak at 5.75 min of CelBP-TAMRA (H-GQVLNPYYSQCK(TAMRA)-NH_2) from 190 nm - 700 nm. Use of the HPLC analytical method described in the paper. Injection volume = 10 µL, ß = 200 µg/mL.

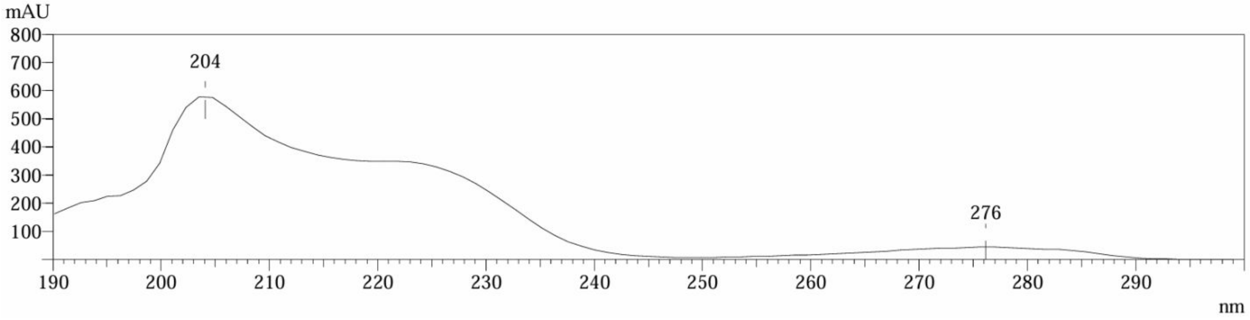

Figure S 64. Absorbance spectrum of the peak of CelBP-N5D (H-GQVLDPYYSQCK-NH_2) from 190 nm - 300 nm. Use of the HPLC analytical method described in the paper. Injection volume = 10 µL, ß = 200 µg/mL.

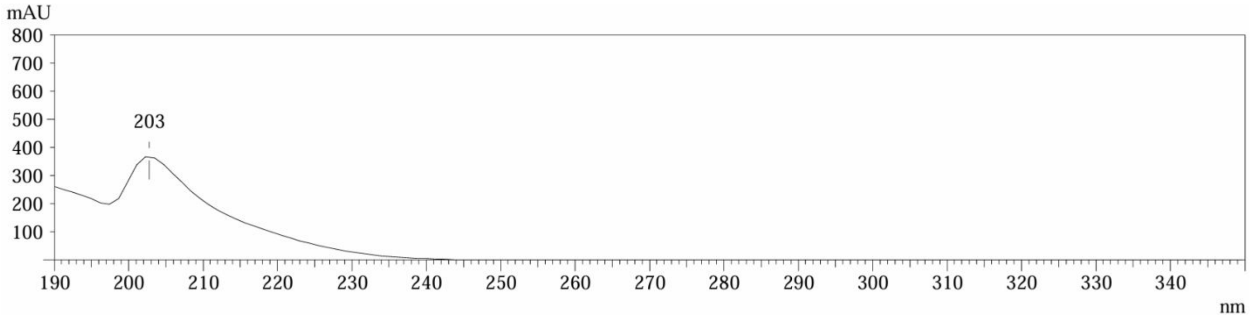

Figure S 65. Absorbance spectrum of the peak at 5.65 min of SM1 (H-GSITQGIPR-NH_2) from 190 nm - 350 nm. Use of the HPLC analytical method described in the paper. Injection volume = 10 µL, ß = 200 µg/mL.

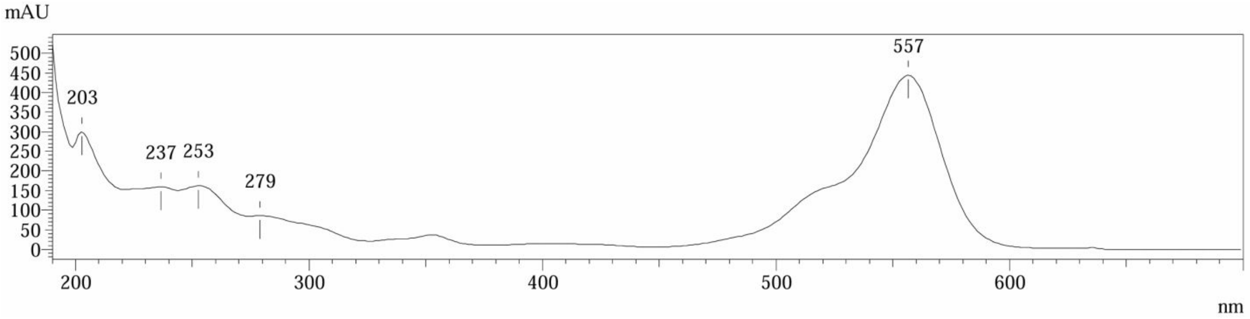

Figure S 66. Absorbance spectrum of the peak at 5.65 min of TAMRA-SM1 (TAMRA-GSITQGIPR-NH_2) from 190 nm - 700 nm. Use of the HPLC analytical method described in the paper. Injection volume = 10 µL, ß = 200 µg/mL.

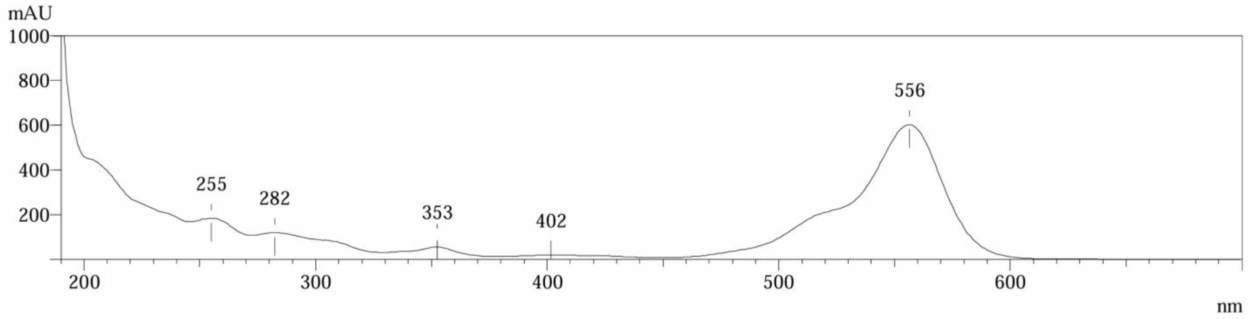

Figure S 67. Absorbance spectrum of the peak at 5.84 min of TAMRA-SM1 (TAMRA-GSITQGIPR-NH_2) from 190 nm - 700 nm. Use of the HPLC analytical method described in the paper. Injection volume = 10 µL, ß = 200 µg/mL.

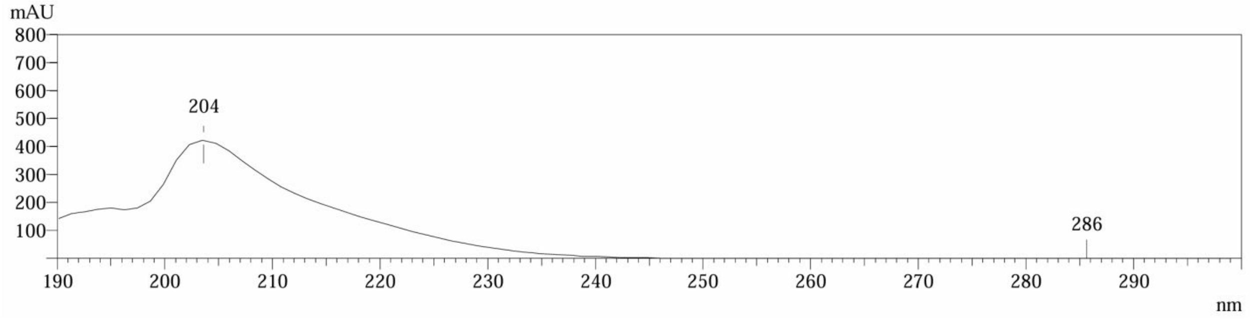

Figure S 68. Absorbance spectrum of the peak of SM1-R9A (H-GSITQGIPA-NH_2) from 190 nm - 300 nm. Use of the HPLC analytical method described in the paper. Injection volume = 10 µL, ß = 200 µg/mL.

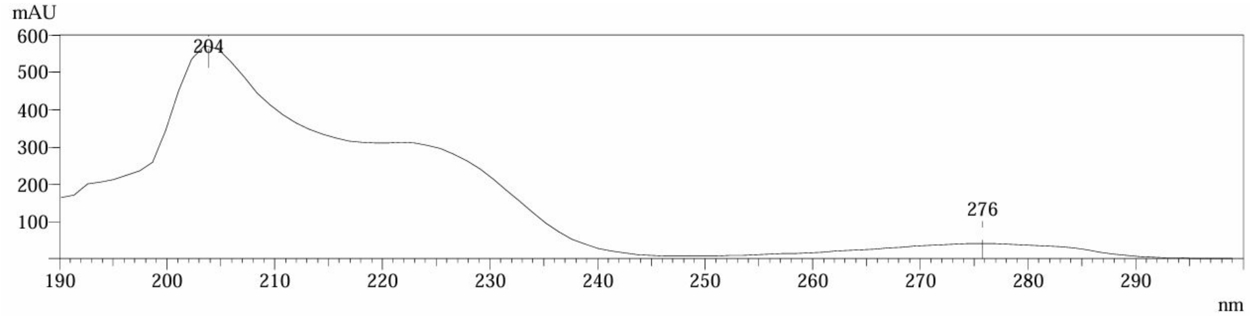

Figure S 69. Absorbance spectrum of the peak of AAP (H-RAYVVM-NH_2) from 190 nm – 300 nm. Use of the HPLC analytical method described in the paper. Injection volume = 10 µL, ß = 200 µg/mL.

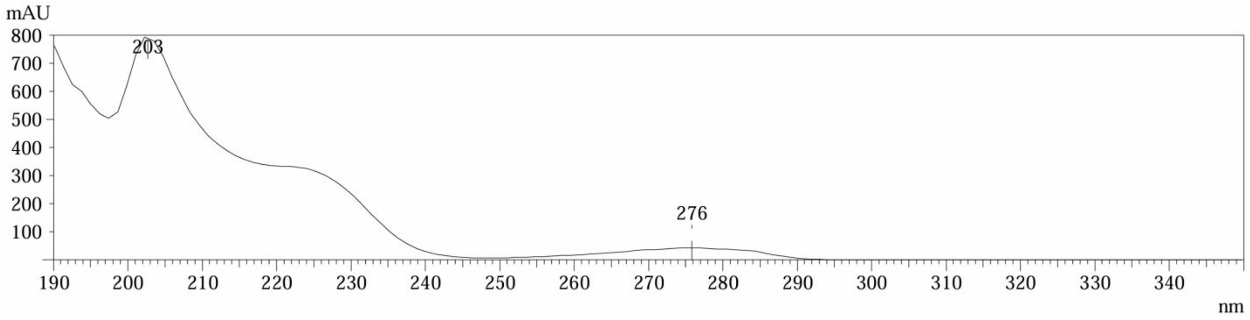

Figure S 70. Absorbance spectrum of the peak of AAP-OH (H-RAYVVM-OH) from 190 nm – 350 nm. Use of the HPLC analytical method described in the paper. Injection volume = 10 µL, ß = 200 µg/mL.

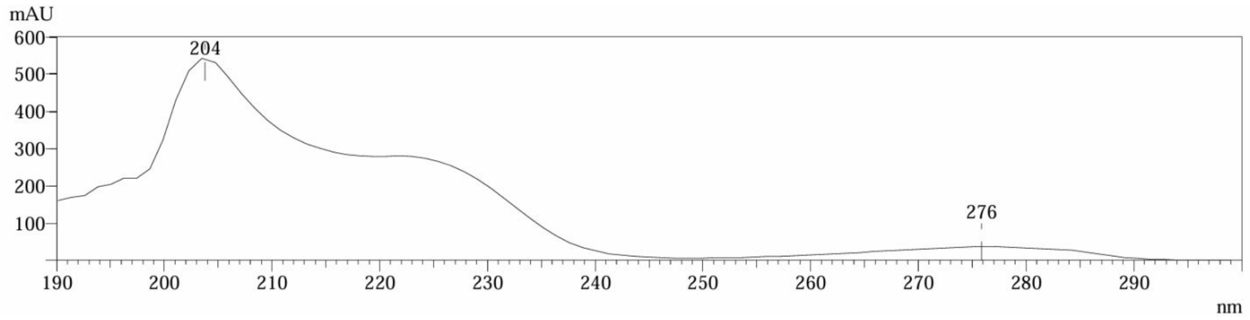

Figure S 71. Absorbance spectrum of the peak of Ac-AAP (Ac-RAYVVM-NH_2) from 190 nm – 300 nm. Use of the HPLC analytical method described in the paper. Injection volume = 10 µL, ß = 200 µg/mL.

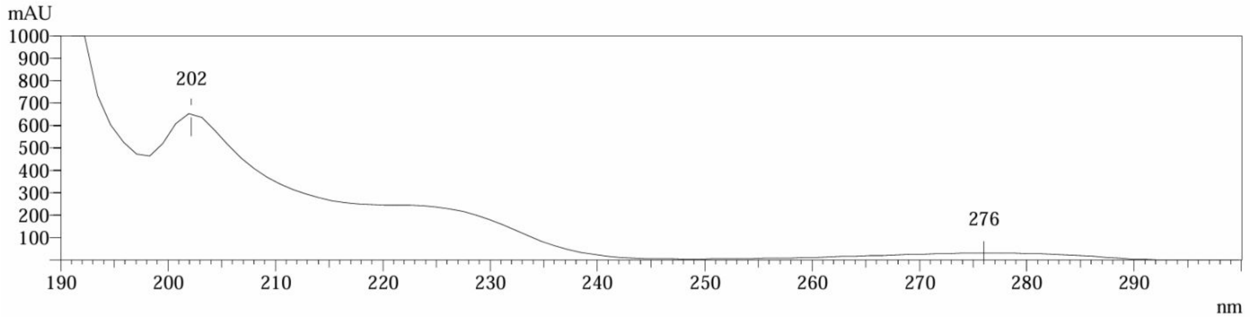

Figure S 72. Absorbance spectrum of the peak of AAP-R1A (H-AAYVVM-NH_2) from 190 nm - 300 nm. Use of the HPLC analytical method described in the paper. Injection volume = 10 µL, ß = 200 µg/mL.

5. Overview of purities, retention times and absorption maxima of the peptides

The described purities in Table 1 were determined by HPLC.

Table 1. Overview of purities, retention times and absorption maxima of the peptides. Values determined by HPLC with UV/Vis detection. The analytical method used is described in the paper. Purities were determined at 220 nm. For TAMRA-labeled peptides, the peak areas of both regioisomers were added and used to calculate purity.

peptide	Sequence	purity (%)	retention time (min)	absorption maximum (nm)
PDDP	H-SQTLYAR-NH_2	99.9	4.51	203
PDDP-Y5S	H-SQTLSAR-NH_2	100.0	4.22	203
PDDP-Y5A	H-SQTLAAR-NH_2	98.3	4.29	204
TAMRA-PDDP	TAMRA-SQTLYAR-NH_2	95.2	5.51/5.75	557/558
PDDP-OH	H-SQTLYAR-OH	99.8	4.54	203
Ac-PDDP	Ac-SQTLYAR-NH_2	90.0	4.73	203
CelBP	H-GQVLNPYYSQCK-NH_2	96.3	4.87	201
CelBP-TAMRA	H-GQVLNPYYSQCK (TAMRA)-NH_2	93.3	5.67/5.80	557
CelBP-N5D	H-GQVLDPYYSQCK-NH_2	99.7	4.96	204
SM1	H-GSITQGIPR-NH_2	94.8	4.71	203
TAMRA-SM1	TAMRA-GSITQGIPR-NH_2	91.0	6.65/5.84	556/557
SM1-R9A	H-GSITQGIPA-NH_2	100.0	4.79	204
AAP	H-RAYVVM-NH_2	93.2	5.06	204

AAP-OH	H-RAYVVM-OH	100.0	5.23	203
Ac-AAP	Ac-RAYVVM-NH_2	79.2	5.35	204

6. CD spectra of the purified peptides

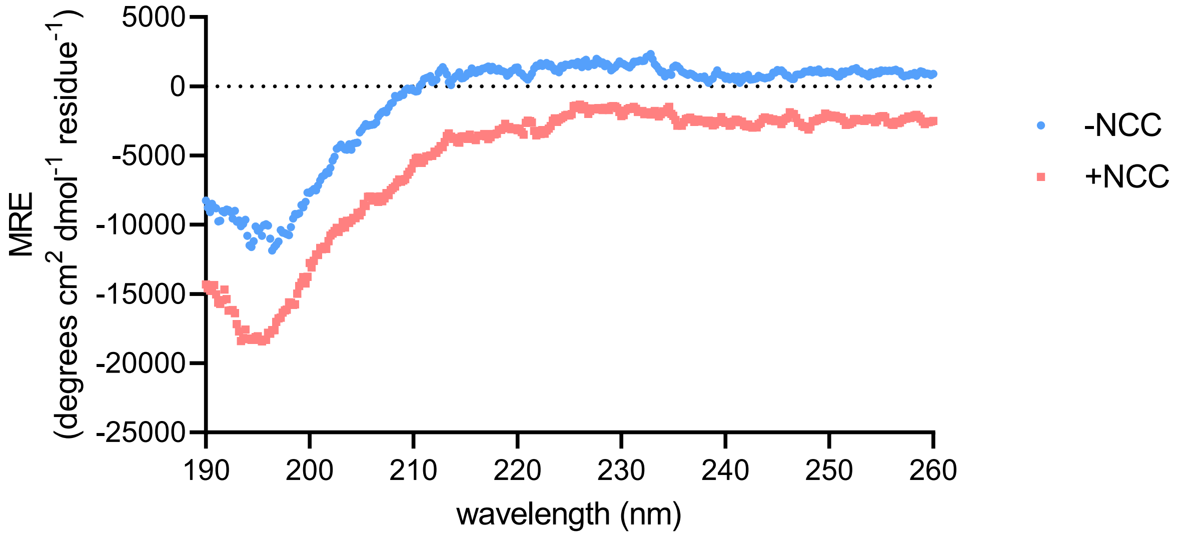

Figure S 73. CD spectrum of PDDP (H-SQTLYAR-NH_2) in ultrapure water (c = 25 µM). Measured with (red squares) and without NCC (8 µM) (blue dots). Measuring temperature: 20˚C, wavelength range from 190 nm - 260 nm.

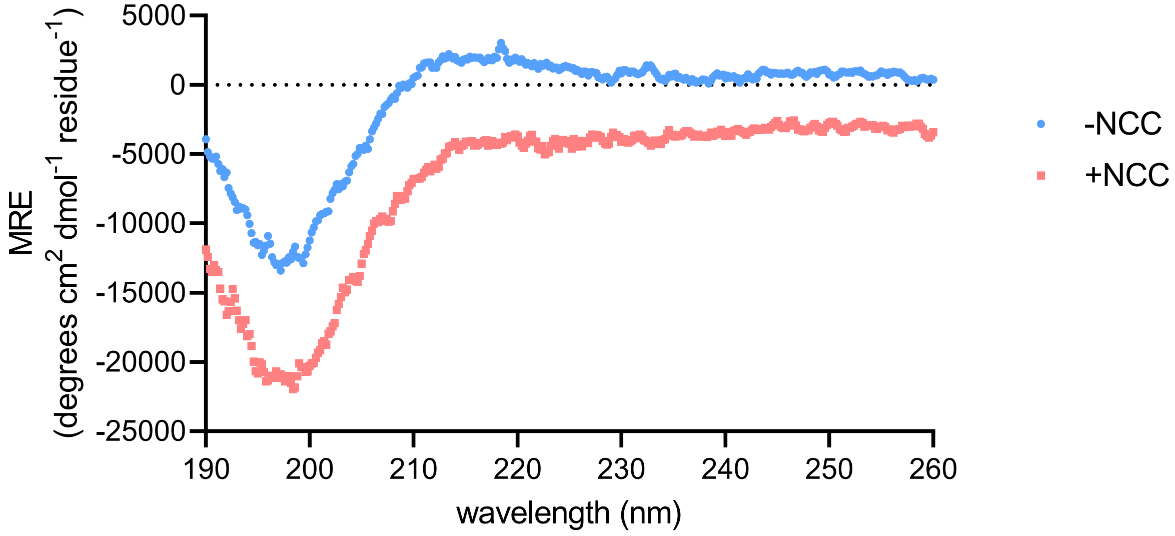

Figure S 74. CD spectrum of PDDP-Y5S (H-SQTLSAR-NH_2) in ultrapure water (c = 25 µM). Measured with (red squares) and without NCC (8 µM) (blue dots). Measuring temperature: 20˚C, wavelength range from 190 nm - 260 nm.

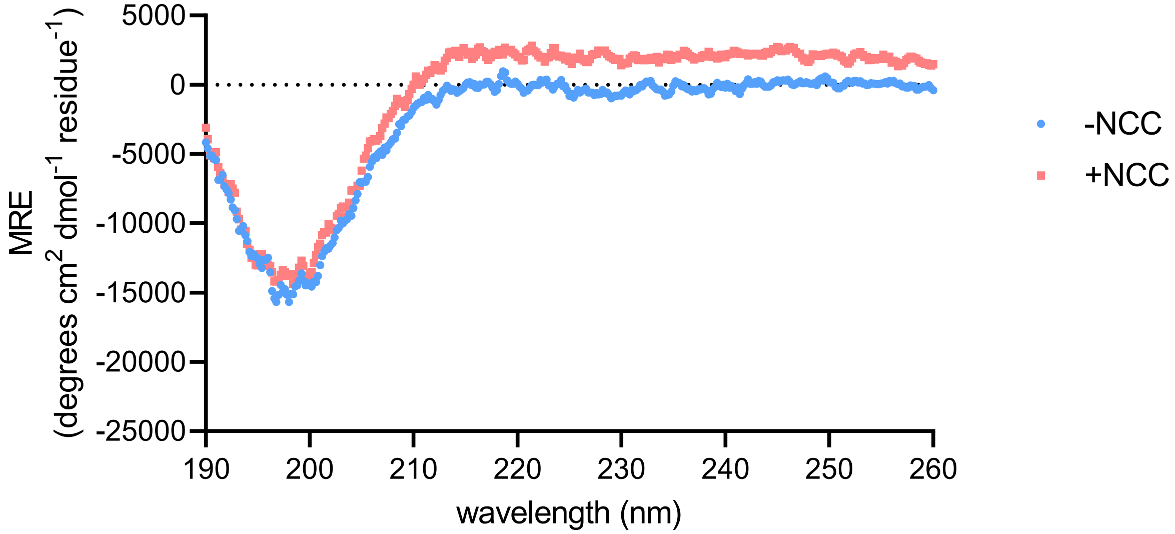

Figure S 75. CD spectrum of PDDP-Y5A (H-SQTLAAR-NH_2) in ultrapure water (c = 25 µM). Measured with (red squares) and without NCC (8 µM) (blue dots). Measuring temperature: 20˚C, wavelength range from 190 nm - 260 nm.

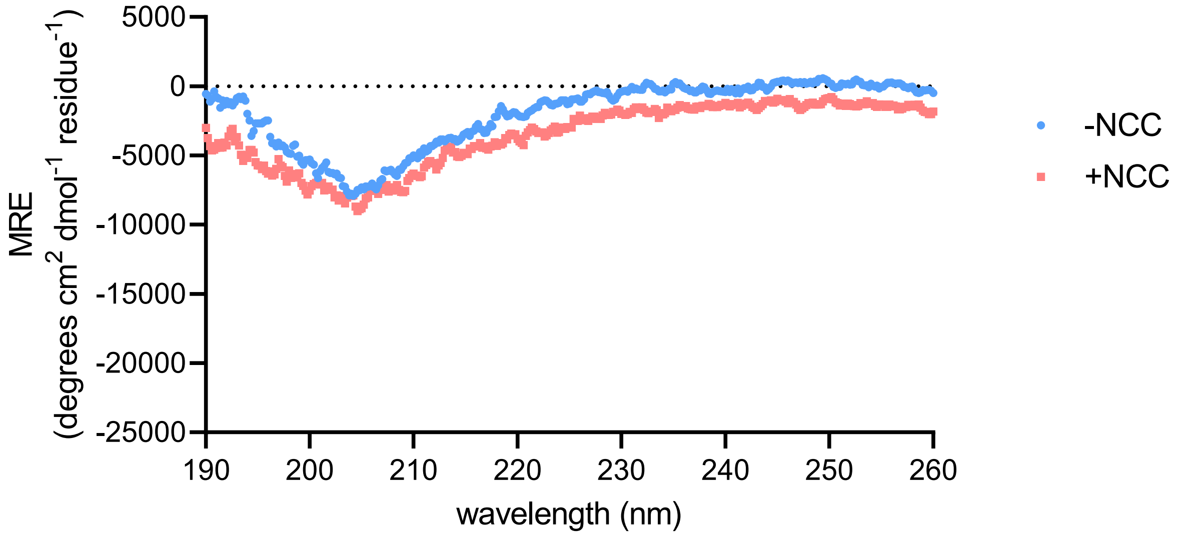

Figure S 76. CD spectrum of TAMRA-PDDP (TAMRA-SQTLYAR-NH_2) in ultrapure water (c = 25 µM). Measured with (red squares) and without NCC (8 µM) (blue dots). Measuring temperature: 20˚C, wavelength range from 190 nm - 260 nm.

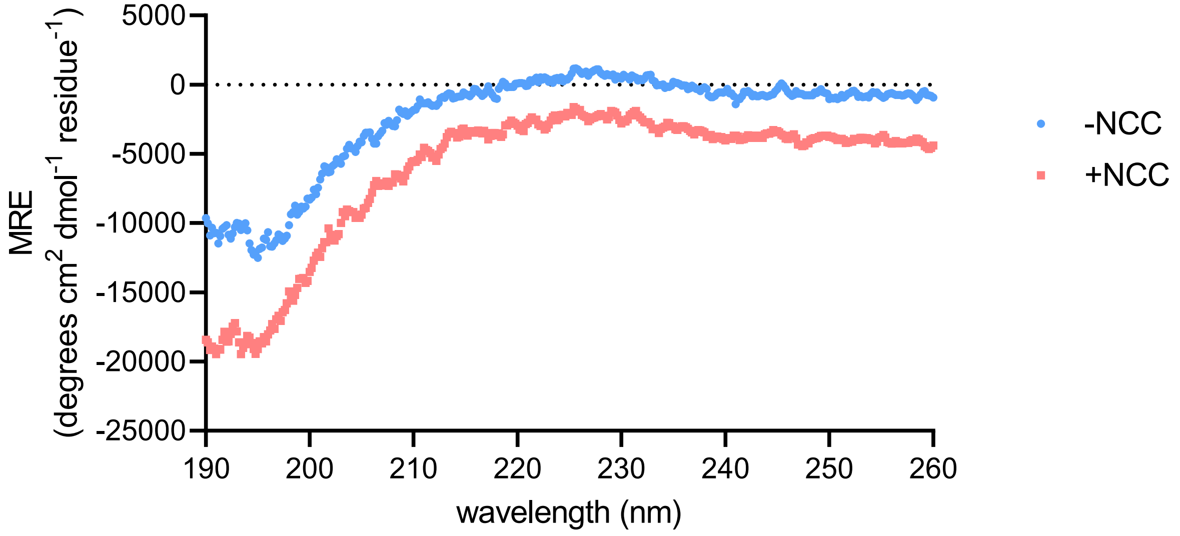

Figure S 77. CD spectrum of PDDP-OH (H-SQTLYAR-OH) in ultrapure water (c = 25 µM). Measured with (red squares) and without NCC (8 µM) (blue dots). Measuring temperature: 20˚C, wavelength range from 190 nm - 260 nm.

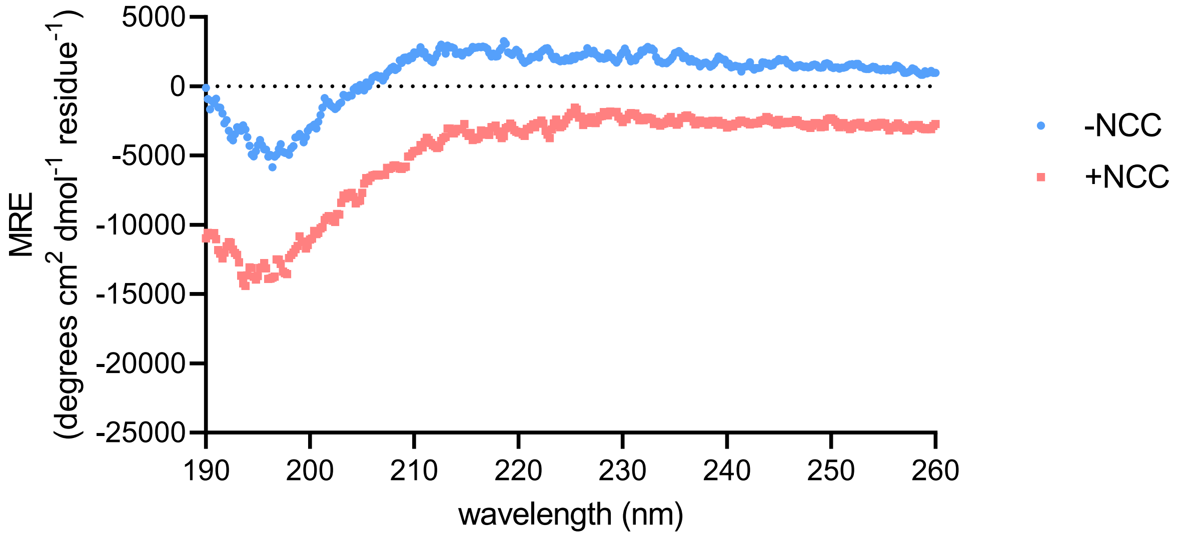

Figure S 78. CD spectrum of Ac-PDDP (Ac-SQTLYAR-NH_2) in ultrapure water (c = 25 µM). Measured with (red squares) and without NCC (8 µM) (blue dots). Measuring temperature: 20˚C, wavelength range from 190 nm - 260 nm.

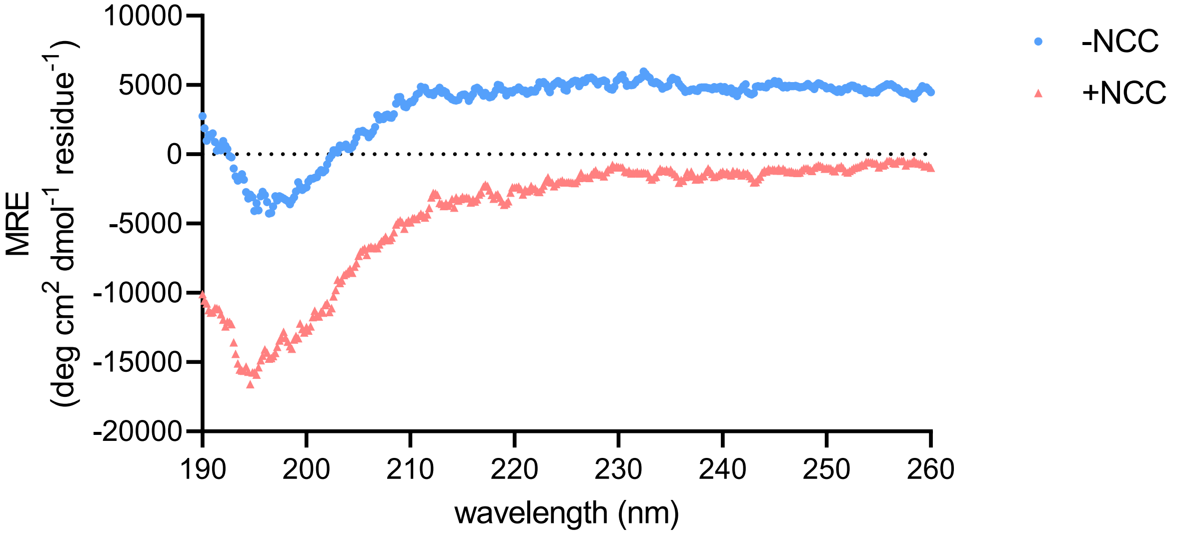

Figure S 79. CD spectrum of PDDP-R7A (H-SQTLYAA-NH_2) in ultrapure water (c = 25 µM). Measured with (red squares) and without NCC (8 µM) (blue dots). Measuring temperature: 20˚C, wavelength range from 190 nm - 260 nm.

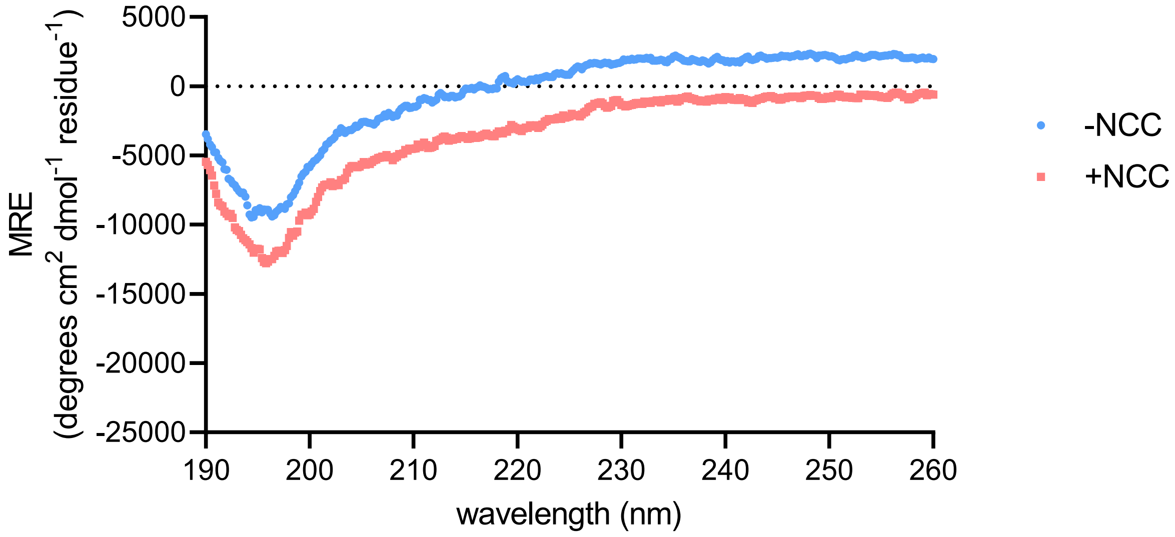

Figure S 80. CD spectrum of CelBP (H-GQVLNPYYSQCK-NH_2) in ultrapure water (c = 25 µM). Measured with (red squares) and without NCC (8 µM) (blue dots). Measuring temperature: 20°C, wavelength range from 190 nm - 260 nm.

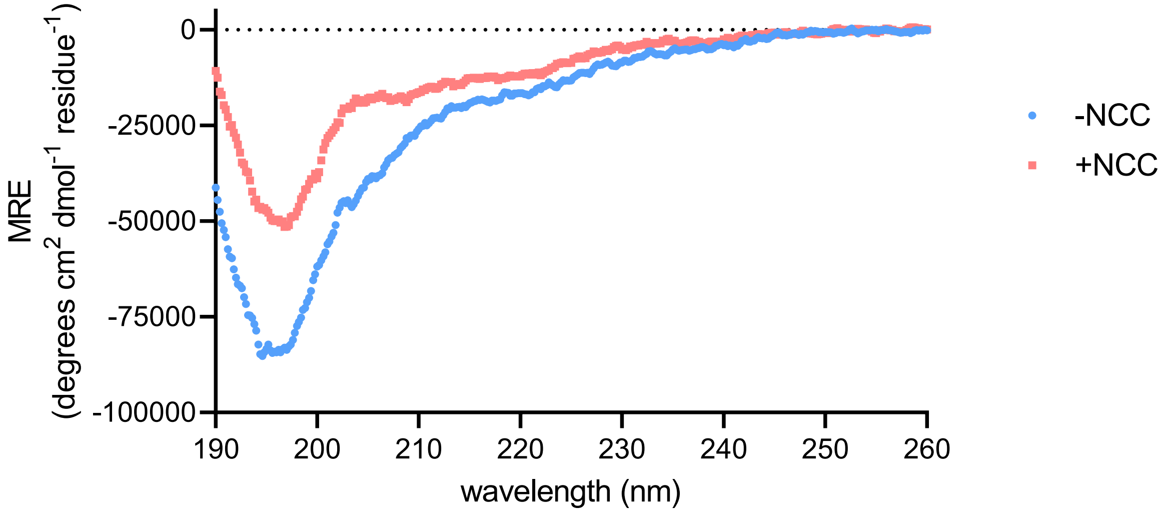

Figure S 81. CD spectrum of CelBP-TAMRA (H-GQVLNPYYSQCK(TAMRA)-NH_2) in ultrapure water (c = 25 µM). Measured with (red squares) and without NCC (8 µM) (blue dots). Measuring temperature: 20°C, wavelength range from 190 nm - 260 nm.

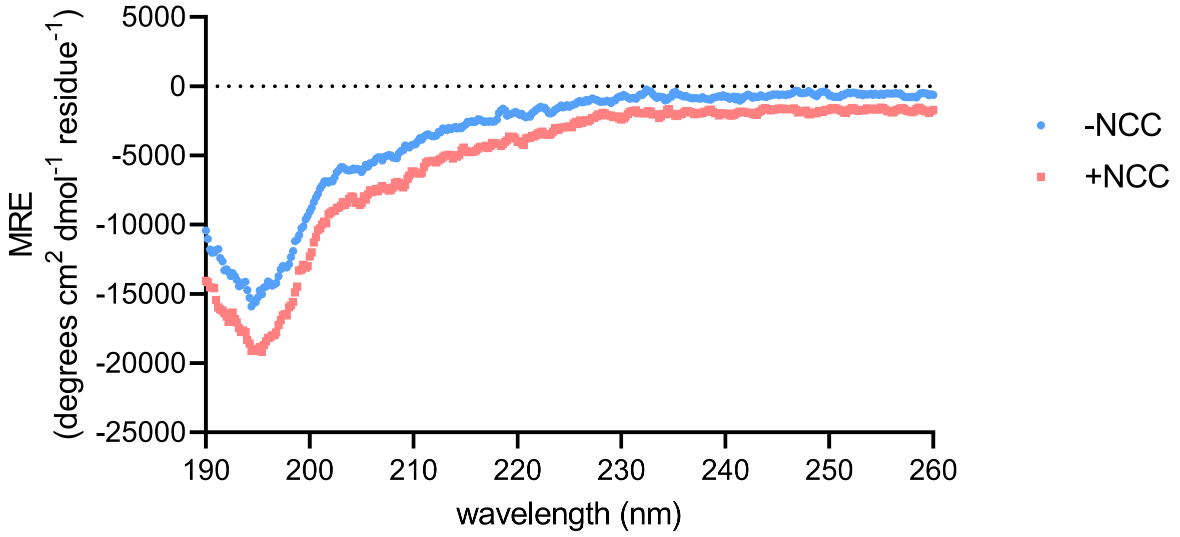

Figure S 82. CD spectrum of CelBP-N5D (H-GQVLDPYYSQCK-NH_2) in ultrapure water (c = 25 µM). Measured with (red squares) and without NCC (8 µM) (blue dots). Measuring temperature: 20˚C, wavelength range from 190 nm - 260 nm.

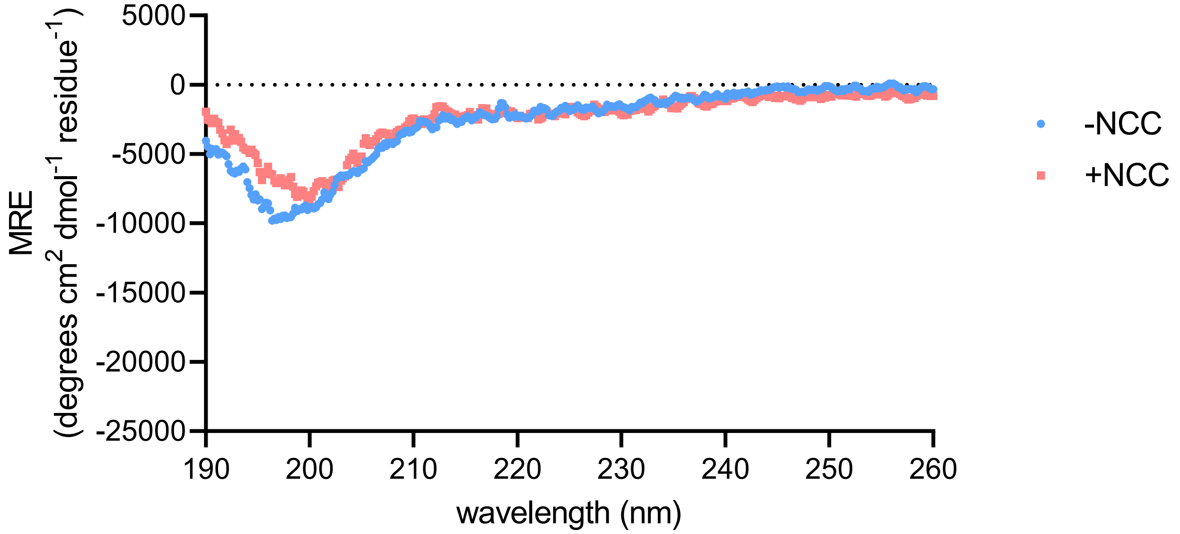

Figure S 83. CD spectrum of SM1 (H-GSITQGIPR-NH_2) in ultrapure water (c = 25 µM). Measured with (red squares) and without NCC (8 µM) (blue dots). Measuring temperature: 20˚C, wavelength range from 190 nm - 260 nm.

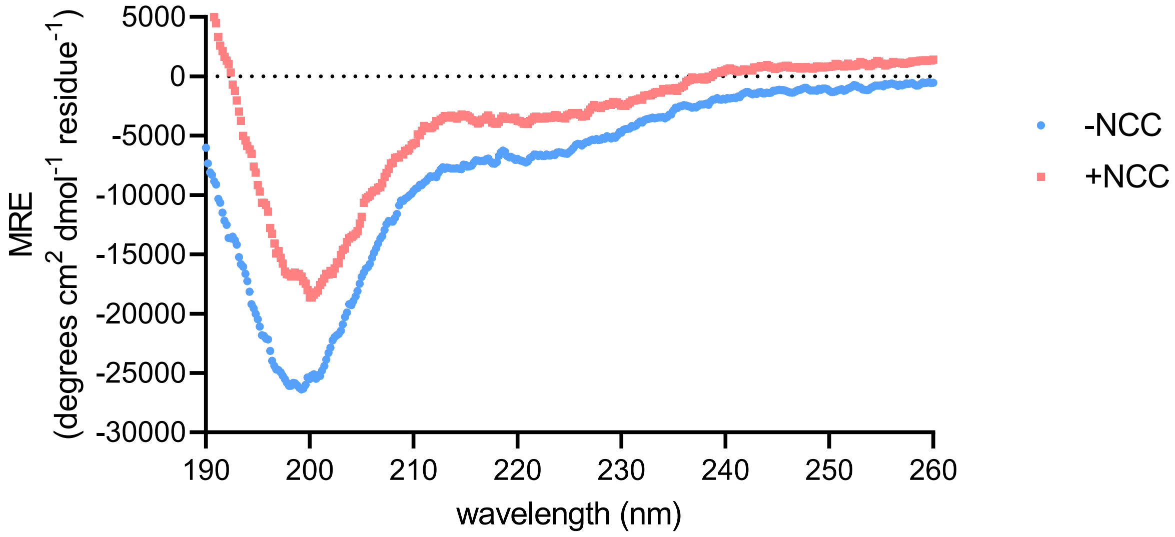

Figure S 84. CD spectrum of TAMRA-SM1 (TAMRA-GSITQGIPR-NH_2) in ultrapure water (c = 25 µM). Measured with (red squares) and without NCC (8 µM) (blue dots). Measuring temperature: 20˚C, wavelength range from 190 nm - 260 nm.

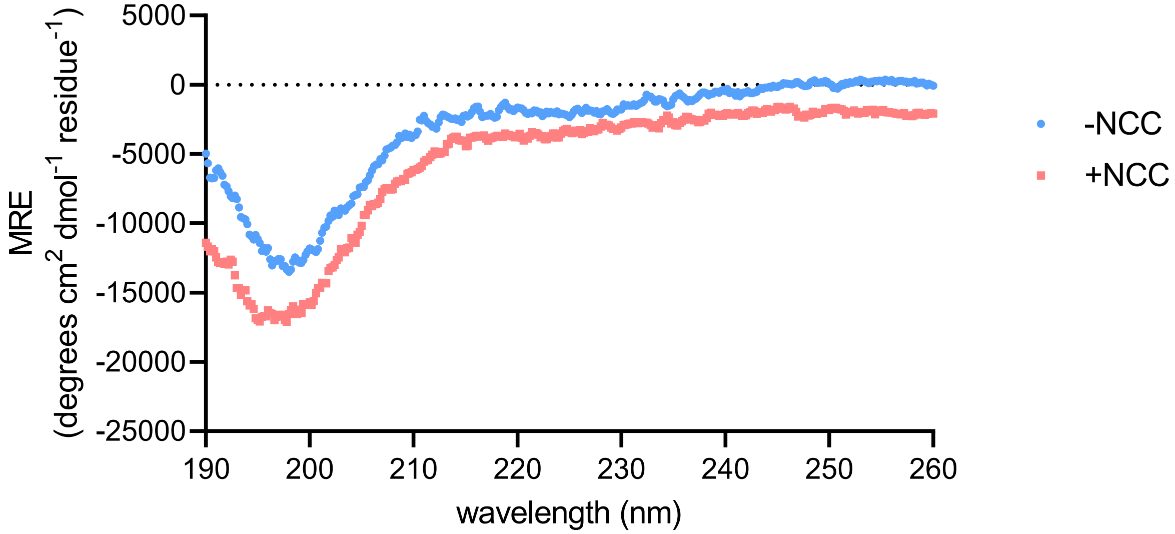

Figure S 85. CD spectrum of SM1-R9A (H-GSITQGIPA-NH_2) in ultrapure water (c = 25 µM). Measured with (red squares) and without NCC (8 µM) (blue dots). Measuring temperature: 20˚C, wavelength range from 190 nm - 260 nm.

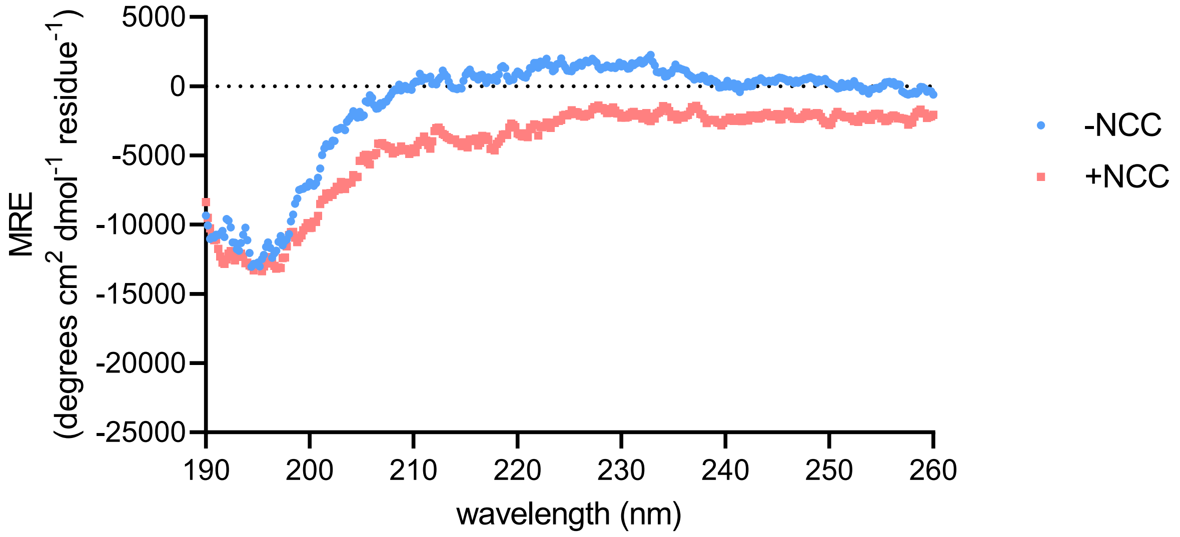

Figure S 86. CD spectrum of AAP (H-RAYVVM-NH_2) in ultrapure water (c = 25 µM). Measured with (red squares) and without NCC (8 µM) (blue dots). Measuring temperature: 20˚C, wavelength range from 190 nm - 260 nm.

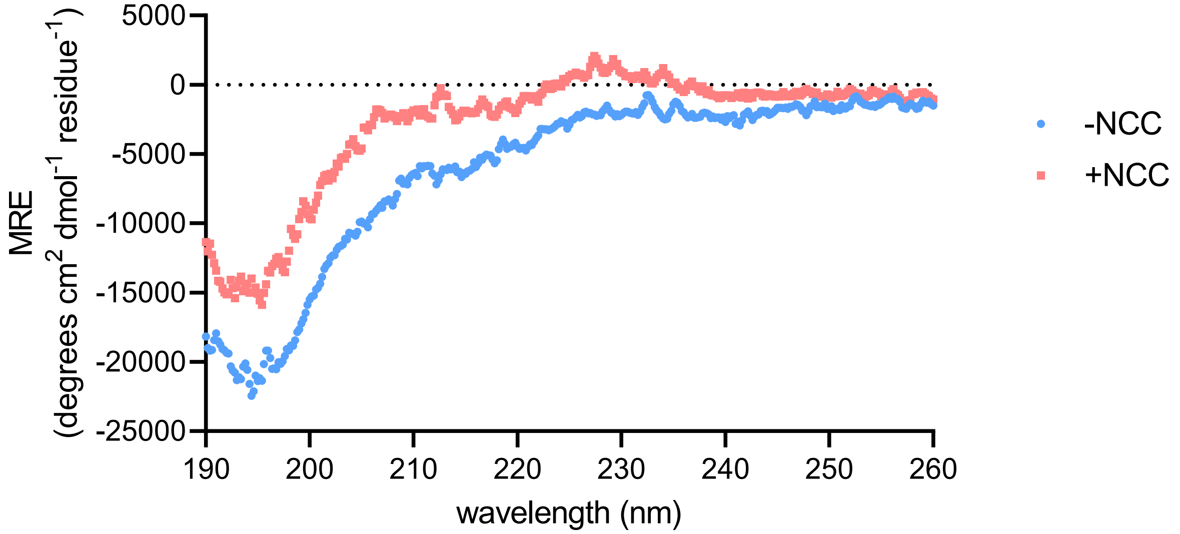

Figure S 87. CD spectrum of AAP-OH (H-RAYVVM-OH) in ultrapure water (c = 25 µM). Measured with (red squares) and without NCC (8 µM) (blue dots). Measuring temperature: 20˚C, wavelength range from 190 nm - 260 nm.

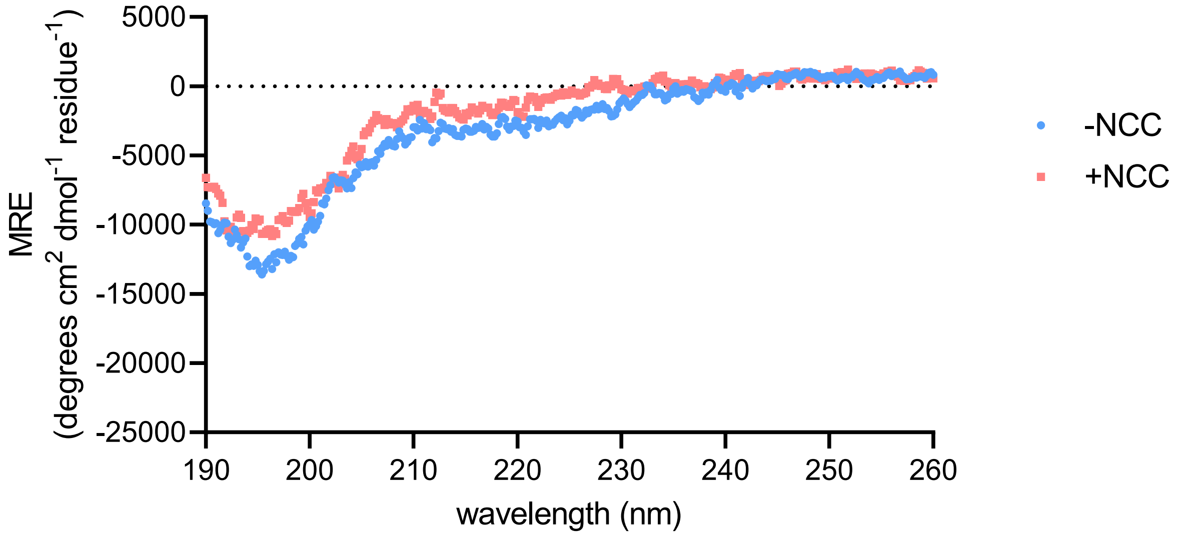

Figure S 88. CD spectrum of Ac-AAP (Ac-RAYVVM-NH_2) in ultrapure water (c = 25 µM). Measured with (red squares) and without NCC (8 µM) (blue dots). Measuring temperature: 20˚C, wavelength range from 190 nm - 260 nm.

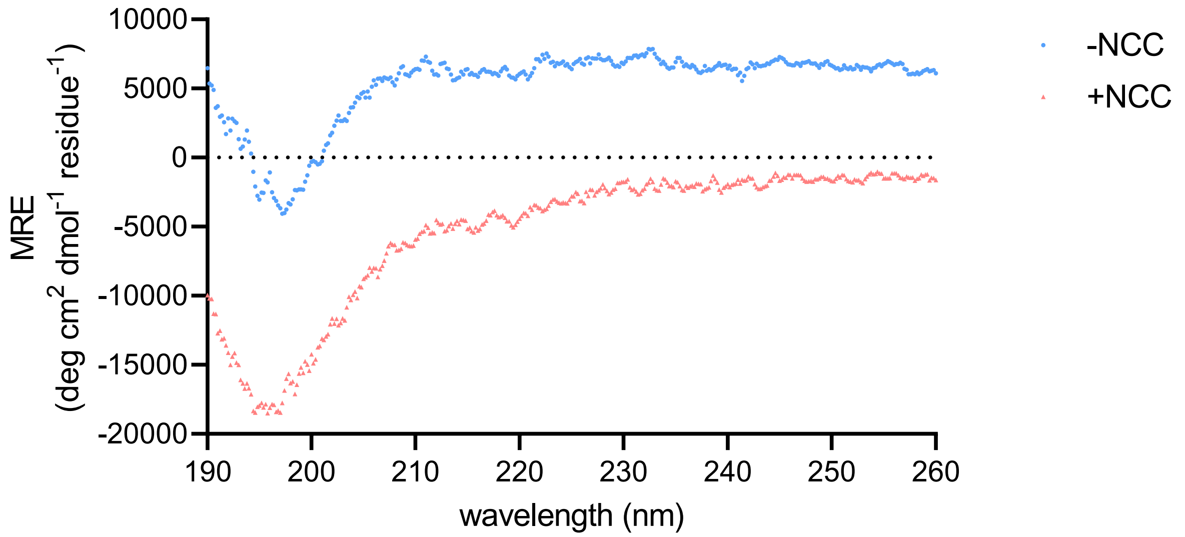

Figure S 89. CD spectrum of AAP-R1A (H-AAYVVM-NH_2) in ultrapure water (c = 25 µM). Measured with (red squares) and without NCC (8 µM) (blue dots). Measuring temperature: 20˚C, wavelength range from 190 nm - 260 nm.
